# Role of Intron-Mediated Enhancement on Accumulation of an *Arabidopsis* NB-LRR Class R*-*protein that Confers Resistance to *Cucumber mosaic virus*

**DOI:** 10.1371/journal.pone.0099041

**Published:** 2014-06-10

**Authors:** Yukiyo Sato, Sugihiro Ando, Hideki Takahashi

**Affiliations:** Department of Applied Plant Science, Graduate School of Agricultural Science, Tohoku University, Sendai, Miyagi, Japan; The Ohio State University, United States of America

## Abstract

The accumulation of RCY1 protein, which is encoded by *RESISTANCE TO CMV(Y)* (*RCY1*), a CC-NB-LRR class *R*-gene, is tightly correlated with the strength of the resistance to a yellow strain of *Cucumber mosaic virus* [CMV(Y)] in *Arabidopsis thaliana*. In order to enhance resistance to CMV by overexpression of *RCY1*, *A. thaliana* was transformed with intron-less *RCY1* cDNA construct under the control of strong CaMV35S promoter. Remarkably, a relative amount of RCY1 protein accumulation in the transformants was much lower than that in plants expressing genomic *RCY1* under the control of its native promoter. To identify a regulatory element of *RCY1* that could cause such differential levels of RCY1 accumulation, a series of *RCY1* cDNA and genomic *RCY1* constructs were transiently expressed in *Nicotiana benthamiana* leaves by the *Agrobacterium*-mediated infiltration method. Comparative analysis of the level of RCY1 accumulation in the leaf tissues transiently expressing each construct indicated that the intron located in the *RCY1*-coding region of genomic *RCY1*, but not the native *RCY1* genomic promoter or the 5′-and 3′-untranslated regions of *RCY1*, was indispensable for high level RCY1 accumulation. The increased levels of RCY1 accelerated plant disease defense reactions. Interestingly, such intron-mediated enhancement of RCY1 accumulation depended neither on the abundance of the *RCY1* transcript nor on the *RCY1* specific-intron sequence. Taken together, intron-mediated *RCY1* expression seems to play a key role in the expression of complete resistance to CMV(Y) by maintaining RCY1 accumulation at high levels.

## Introduction

Over the past 20 years, more than 80 resistance (*R*) genes that confer resistance to plant pathogens via a defense reaction have been cloned [1]–[3]. Common features of these R proteins include the conserved nucleotide-binding (NB) and leucine-rich-repeat (LRR) domains that they contain [4], [5]. The NB-LRR domains-containing R proteins are further separated into two subclasses depending on whether they have a coiled-coil (CC) domain or a toll-interleukin-1 receptor (TIR)-like region at their amino terminus [6]. To date, 149 NB-LRR-encoding genes (including 55 CC-NB-LRR-encoding and 94 TIR-NB-LRR-encoding genes) and another 58 related genes that do not encode an LRR domain have been identified in the genome sequence of *Arabidopsis thaliana* ecotype Columbia (Col-0) [6]. Nearly half of the NB-LRR-encoding genes are scattered throughout the genome as simple loci, whereas the remaining NB-LRR-encoding genes are located in gene family clusters that might have become duplicated and expanded due to unequal crossing-over between mispaired tandem copies during meiosis [6]–[8].

Although an increased number of NB-LRR-encoding genes and related genes in *Arabidopsis* genome were classified based on protein motifs, intron positions and sequence conservation, our understanding of the regulation of their gene expression is still limited. Global expression analysis of NB-LRR-encoding and related genes in *Arabidopsis* suggests that most of their transcripts are present at low levels [9]. The expression of a limited number of these genes can be induced by the bacterial flagellin peptide, flg22 or by salicylic acid (SA) treatment [9]. However, significant induction of known *R* gene expression during the plant defense response seems to occur only in a minority of the cases examined so far [9]–[15]. The transcript levels of other *R* genes do not change in response to pathogen attack [16]–[19]. Furthermore, global expression analysis of NB-LRR-encoding and related genes in *Arabidopsis* suggests that most NB-LRR-encoding genes tend to be more responsive to pathogens than randomly selected genes, but less responsive to pathogens than common defense-related marker genes [9], [15]. Therefore, in general, *R* genes may not generally need to be induced at the transcriptional level to regulate resistance to pathogens.

However, for some NB-LRR-encoding genes in *Arabidopsis*, there is evidence of significant correlation between the level of NB-LRR protein accumulation before pathogen infection and enhanced resistance to pathogens [20], [21]. Along similar lines, overexpression of *R* genes often triggers autoactivation of the defense reaction and spontaneous cell death independent of pathogen infection [22]–[24]. Thus, appropriate control of *R* gene expression seems to be important for a fully functional defense system.

Further, alternative splicing has been reported for a certain number of known NB-LRR-encoding *R* genes: e.g., *N*, *L6*, *RPP5*, *RPS4*, *M*, *RAC1*, *Bs4*, and *Mla6* [17], [25]–[32]. Alternative splicing is known to be a requirement for the function of both of the plant R protein *N* and *RPS4*, govern the resistance to *Tobacco mosaic virus* (TMV) and to *Pseudomonas syringae* pv *tomato* carrying *AvrRPS4*, respectively [29], [33]. For a certain number of NB-LRR-encoding genes, alternative splicing may be indispensable for complete resistance to pathogens, although the mechanism and role of alternative splicing in NB-LRR-encoding gene-mediated disease resistance remains to be understood [34]. Overall, the regulatory system for controlling *R* gene expression requires much further analysis.

We have previously demonstrated that *RESISTANCE TO CMV(Y)* (*RCY1*), which encodes a CC-NB-LRR class resistance protein in *A. thaliana*, confers the resistance to a yellow strain of *Cucumber mosaic virus* [CMV(Y)] in an RCY1 protein accumulation-dependent manner [21], [24], [35]–[36]. *RCY1*-mediated resistance to CMV(Y) is accompanied by the development of necrotic local lesions at the primary infection sites, elevated expression of defense-related genes, such as *Pathogenesis-Related 1a* (*PR-1a*), and accumulation of SA [37], [38]. Interestingly, increased expression of *RCY1* converts the hypersensitive resistance response (HR) to an extreme resistance response (ER) to CMV(Y) [21], suggesting that the level of RCY1 protein regulates the strength of resistance to CMV(Y) in *Arabidopsis*. Therefore, analysis of the mechanisms of regulation of *RCY1* gene expression should provide new approaches to modulate NB-LRR-class R-protein-mediated defense reaction against pathogen attack. In this study, we identified a genomic element of *RCY1* that enhances *RCY1*-conferred resistance to CMV(Y) by elevating level of RCY1 accumulation in *A. thaliana* and *N. benthamiana*.

## Results

### Detection of *RCY1* transcript in CMV(Y)-inoculated leaves of *Arabidopsis thaliana*

**Figure 1:**
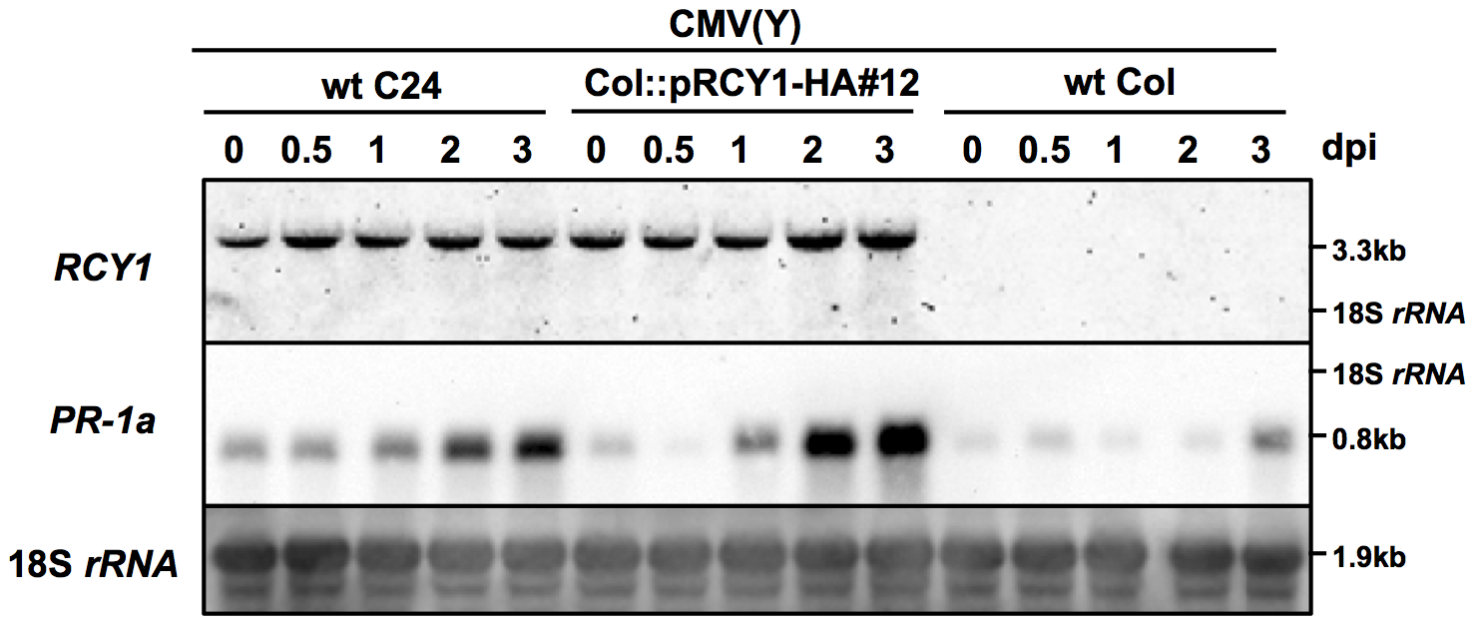
Detection of *RCY1* and *Pathogenesis-Related 1a* transcripts in *Arabidopsis thaliana* ecotypes and *RCY1*-transformants. *RESISTANCE TO CMV(Y)* (*RCY1*) and *Pathogenesis-Related 1a* (*PR-1a*) transcripts in CMV(Y)-inoculated leaves of wild-type *A. thaliana* ecotypes C24 (wt C24) and Col-0 (wt Col) and HA epitope-tagged *RCY1*-transformed Col-0 (Col::pRCY1-HA#12) were detected by northern hybridization at 0, 0.5, 1, 2, and 3 days post-inoculation (dpi). As an internal control for RNA sample loading, 18S *rRNA* is shown. The size of each band and the position of 18S *rRNA* were shown at right side of the panel. For all experiments, three independent plants per line were used and a photograph of a representative plant is shown.

*RCY1* transcript was detected in the resistant ecotype C24 carrying *RCY1*, but not in the control susceptible ecotype Columbia-0 (Col-0) without *RCY1*, by northern hybridization with a DIG-labeled *RCY1* DNA probe (Figure 1). The amount of *RCY1* transcript did not significantly increase during the progress of the HR in CMV(Y)-inoculated C24 leaves (Figure 1). *RCY1* transgene transcripts could also be detected in Col-0 transformed with genomic *RCY1* tagged with hemagglutinin (HA)-epitope sequence at its 3′-end (Col::pRCY1-HA#12) [21]. However, the level of *RCY1* transgene expression did not change in CMV(Y)-inoculated leaves of Col::pRCY1-HA#12 as much as in CMV(Y)-inoculated C24 (Figure 1), in contrast, the expression of *PR-1a* a typical marker gene for the HR induction, was clearly up-regulated in CMV(Y)-inoculated leaves of both C24 and Col::pRCY1-HA#12 (Figure 1), suggesting that the expression of neither endogenous nor transformed *RCY1* is inducible in response to CMV(Y) infection. Therefore, the induction of *RCY1* expression alone seems to be insufficient to activate the plant defense system against CMV(Y) in *A. thaliana*.

### Expression of genomic *RCY1* and CaMV35S promoter-driven *RCY1* cDNA transgenes in *A. thaliana*

**Figure 2:**
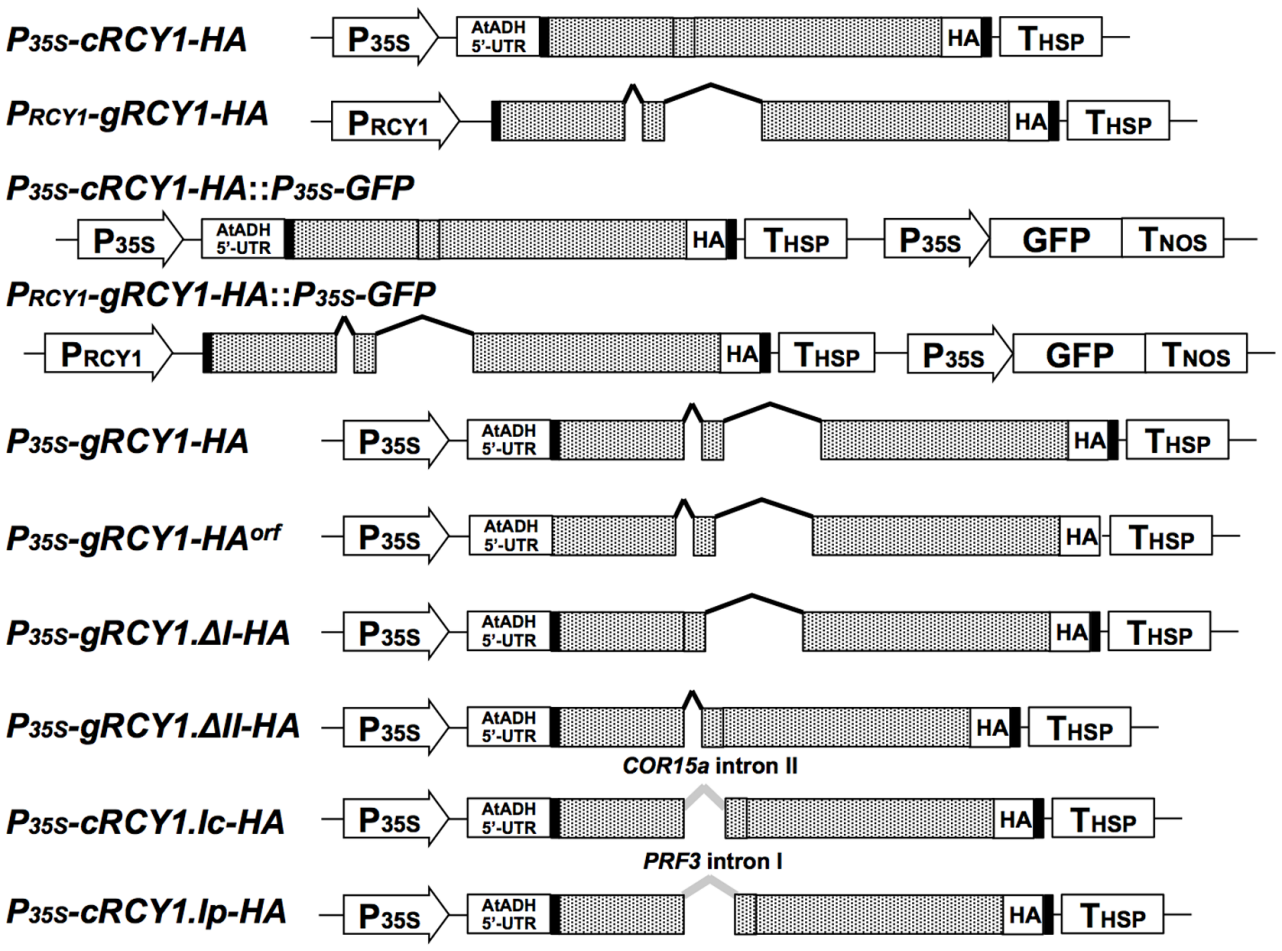
Schematic structure of vector constructs encoding RCY1. *P_35S_-cRCY1-HA* contains the *RCY1* cDNA with no introns but including an HA-epitope tag (HA) at its 3′-end, the 66-bp 5′ sequence upstream of the *RCY1* start codon of (66-bp 5′-UTR), and the 71-bp 3′-downstream sequence from the *RCY1* stop codon (71-bp 3′-UTR). All elements were cloned between the CaMV 35S promoter (P_35S_) and the 5′-UTR sequence of the *Arabidopsis Alcohol Dehydrogenase* gene (ADH5′-UTR) and terminator sequence of the *Heat Shock Protein* gene (T_HSP_) in the pRI201-AN binary vector. *P_RCY1_-gRCY1-HA*, contains the 1.5 kb genomic *RCY1* promoter (P_RCY1_), the genomic *RCY1*-coding regions including two introns and tagged with HA at its 3′-end, and the *RCY1* 66-bp 5′-UTR region and the 71-bp 3′-UTR cloned into the pRI201-AN binary vector. *P_35S_-gRCY1-HA::P_35S_-GFP* and *P_RCY1_-gRCY1-HA::P_35S_-GFP* include P_35S_, the GFP-coding region, and the *nos* terminator (T_NOS_) cloned into the vector pRI201-AN. *P_35S_-gRCY1-HA*, includes the genomic *RCY1*-coding region with two introns tagged with HA at its 3′-end, and the *RCY1* 66-bp 5′-UTR and 71-bp 3′-UTR, cloned downstream of P_35S_ between ADH5′-UTR and T_HSP_ in pRI201-AN. *P_35S_-gRCY1-HA^orf^* was constructed by deletion of 66-bp-5′-UTR and 71-bp-3′-UTR from *P_35S_-gRCY1-HA*. *P_35S_-gRCY1.ΔI-HA* and *P_35S_-gRCY1.ΔII-HA* were constructed by deletion of the first or second intron from *P_35S_-gRCY1-HA*, respectively. *P_35S_-cRCY1.Ic-HA* was constructed by insertion of the *COLD-REGULATED 15A* (*COR15a*) intron II into the first splice junction site in *P_35S_-cRCY1-HA*. *P_35S_-cRCY1.Ip-HA* was created by insertion of the *PROFILIN 3* (*PRF3*) intron I into the first splice junction site in *P_35S_-cRCY1-HA*. In all vector constructs, the 66-bp 5′-UTR and 71-bp 3′-UTR of *RCY1* are shown as black boxes. The RCY1-coding region is indicated by gray boxes in which the two splice junction sites are indicated by vertical lines in the boxes. The two *RCY1* introns are shown as black dashed lines and the *COR15a* or *PRF3* introns are shown as gray dashed lines.

**Figure 3:**
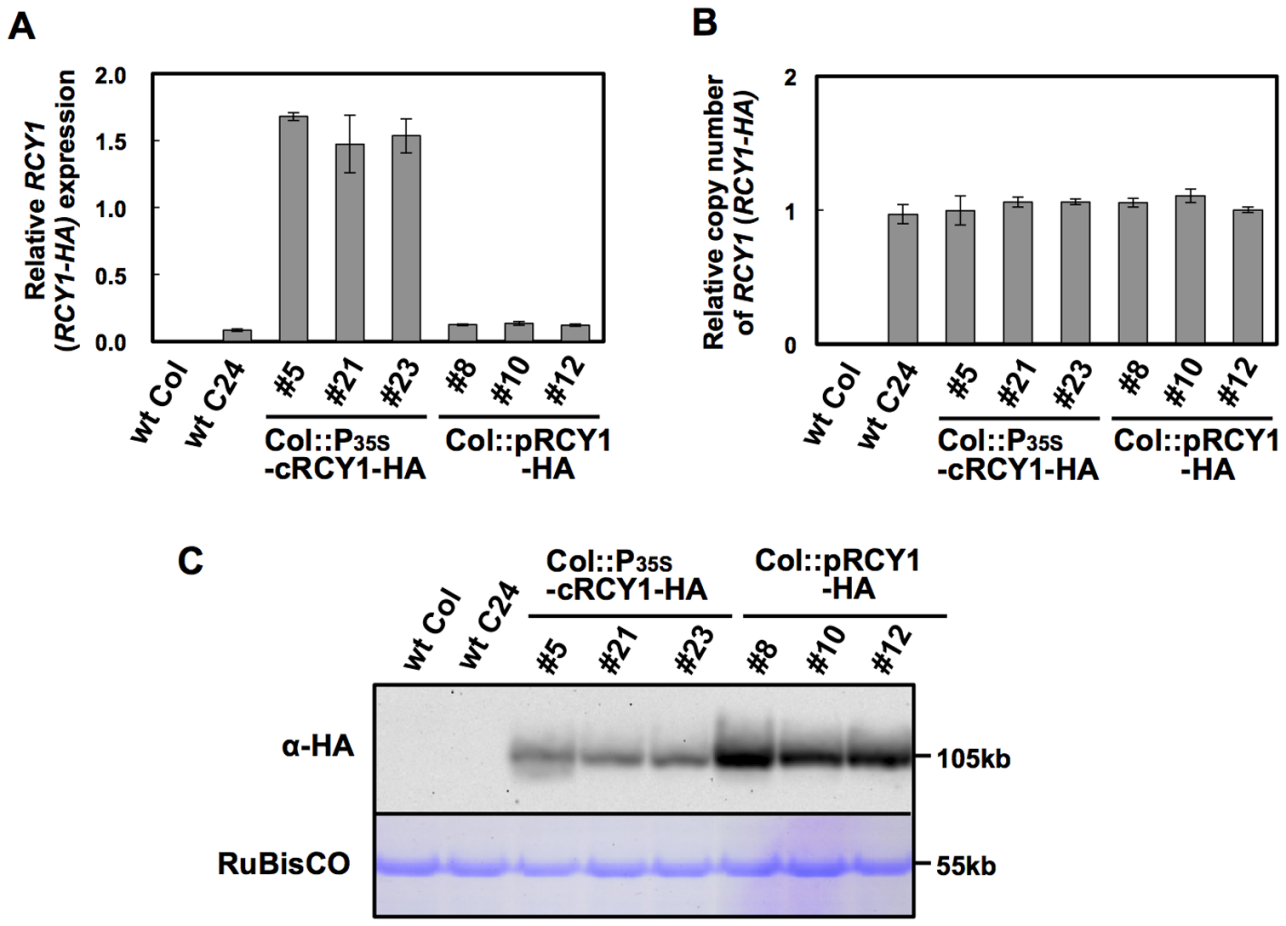
Detection of RCY1 protein, the *RCY1* transcript, and the *RCY1* transgene in three independent Col-0 lines transformed with HA-tagged genomic *RCY1* or HA-tagged *RCY1* cDNA without introns. Relative amounts of *RCY1* transcripts in wild-type *A. thaliana* ecotypes Col-0 (wt Col) and C24 (wt C24), and three independent lines transformed with HA-tagged genomic *RCY1* (Col::pRCY1-HA #8, #10, and #12) or HA-tagged *RCY1* cDNA without introns (Col::cRCY1-HA #5, #21 and #23), were measured by quantitative RT-PCR (A). Relative amounts of RCY1-coding transgene DNA in wild-type ecotypes (wt Col and wt C24) and three independent lines of Col::gRCY1-HA and Col::cRCY1-HA were measured by quantitative PCR using each genomic DNA as a template (B). HA-epitope-tagged RCY1 protein (α-HA) in wild-type ecotypes (wt Col and wt C24); Col::gRCY1-HA #8, #10 and #12 lines; and Col::cRCY1-HA #5, #21 and #23 lines was immunologically detected using monoclonal anti-HA epitope antibody. As an internal control for protein sample quantities, the large subunit of RuBisCO was visualized by staining by Coomassie Brilliant Blue R-250 (CBB) (C). For all experiments, three independent plants per vector were analyzed. The averages of relative *RCY1* transcript amounts ±SE are shown in A and B. In C, a representative photograph is shown. The size of each band was shown at right side of the panel.

We previously found endogenous levels of RCY1 protein accumulation to be correlated with the degree of the resistance to CMV(Y) in *A. thaliana* [21]. Thus, to elevate the basal level of *RCY1* transcript and thereby enhance the resistance to CMV(Y) in *A. thaliana*, we expressed the *P_35S_-cRCY1-HA* construct under the control of 35S promoter of *Cauliflower mosaic virus* (CaMV) in three independent transgenic lines (Col::P_35S_-cRCY1-HA#5, #21 and #23). The *P_35S_-cRCY1-HA* construct consisted of an intron-less *RCY1* cDNA tagged with an HA-epitope sequence at its 3′-end, and included a 66-bp sequence 5′ upstream of the start codon of *RCY1* (66-bp-5′-UTR) and a 71-bp sequence 3′-downstream from the stop codon of *RCY1* (71-bp-3′-UTR) (Figure 2). As a control, three independent lines of Col::pRCY1-HA, #8, #10 and #12, each carrying the intron-containing genomic *RCY1* transgene tagged with an HA-epitope sequence at its 3′-end [21] were grown under the same growth conditions. We assumed that *RCY1* would be expressed at much higher levels in these Col::P_35S_-cRCY1-HA lines than in the Col::pRCY1-HA lines, as the activity of the CaMV35S promoter is generally stronger than that of the native genomic *RCY1* promoter. As expected, the level of *RCY1* transcript in the three independent Col::P_35S_-cRCY1-HA lines was much higher than that in three independent Col::pRCY1-HA lines (Figure 3A). Normalization of PCR-based quantification of genomic *RCY1* or *RCY1* cDNA transgene with *ubiquitin 5* gene (*UBQ5*) suggested that each transgenic line carried the same or similar transgene copy numbers (Figure 3B). Therefore, the increased *RCY1* transcript levels in Col::P_35S_-cRCY1-HA were due not to differential transgene copy numbers in each transgenic lines, but to the strength of the CaMV35S promoter. Next, when the HA-epitope-tagged RCY1 protein was immunologically detected in both transgenic lines using an anti-HA monoclonal antibody, remarkably, the abundance of RCY1 protein in three Col::pRCY1-HA lines was much higher than that in three Col::P_35S_-cRCY1-HA lines (Figure 3C). This inverse relationship between *RCY1* transcript level and RCY1 protein accumulation between genomic *RCY1*-transformed Col::pRCY1-HA lines and *RCY1* cDNA-transformed Col::P_35S_-cRCY1-HA lines could be due to distinctions between the regulatory elements such as intron sequence controlling genomic *RCY1* expression versus *RCY1* cDNA expression.

### Transient expression of genomic *RCY1* and CaMV35S promoter-driven *RCY1* cDNA transgenes in *Nicotiana benthamiana* by *Agrobacterium*-mediated infiltration method

**Figure 4:**
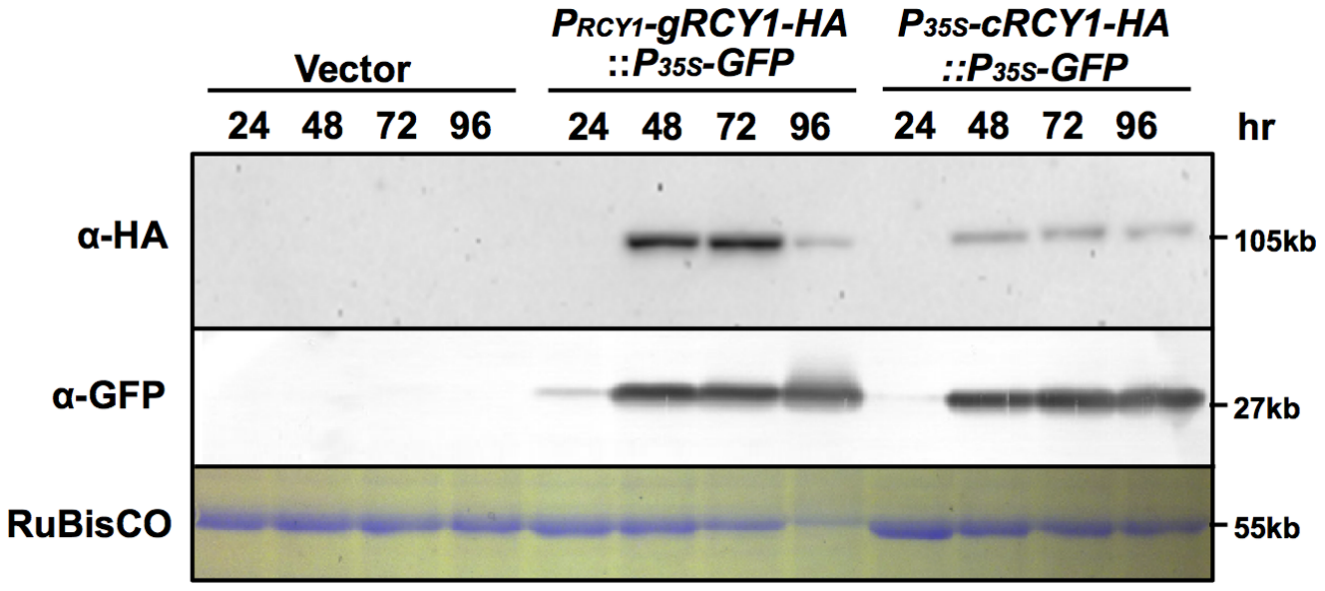
Detection of HA-epitope-tagged RCY1 in *N. benthamiana* leaves transiently expressing genomic *RCY1-HA* under control of the native *RCY1* promoter or the *RCY1* cDNA without introns under control of the CaMV 35S promoter. HA-epitope-tagged RCY1 protein (α-HA) in *N. benthamiana* leaf tissues transiently expressing *P_RCY1_-gRCY1-HA::P_35S_-GFP* or *P_35S_-gRCY1-HA::P_35S_-GFP* was immunologically detected using monoclonal antibody against the HA epitope at 24, 48, 72, and 96 h after agro-infiltration. GFP accumulation (α-GFP) was also immunologically detected at the same time points using polyclonal antibody against GFP as an internal standard. As an internal control for protein sample quantities, the large subunit of RuBisCO was visualized by staining with CBB. The size of each band was shown at right side of the panel. For all experiments, three independent plants per vector were analyzed and representative data are shown.

To further confirm increased RCY1 accumulation upon expression of genomic *RCY1*, genomic *RCY1* tagged with an HA-epitope sequence at its 3′-end was cloned into the *Hin*dIII-*Sal*I sites of pRI201-AN and designated *P_RCY1_-gRCY1-HA* (Figure 2). In *P_RCY1_-gRCY1-HA*, the genomic *RCY1* contains its coding region with two introns, ∼1.5 kbp promoter region and 71-bp 3′-UTR. As an internal control, a DNA fragment containing the Green Fluorescence Protein (GFP)-coding region fused between the CaMV35S promoter (P_35S_) and the nopaline synthase (NOS) terminator (T_NOS_), was inserted downstream of *P_RCY1_-gRCY1-HA* and *P_35S_-cRCY1-HA*, respectively. When these two vector constructs, *P_35S_-cRCY1-HA::P_35S_-GFP* and *P_RCY1_-gRCY1-HA::P_35S_-GFP* (Figure 2), were transiently expressed in *N. benthamiana* leaves by *Agrobacterium*-mediated infiltration (agro-infiltration) method, the level of RCY1 accumulated in leaf tissues expressing *P_RCY1_-gRCY1-HA::P_35S_-GFP* was reproducibly much higher than those expressing *P_35S_-cRCY1-HA::P_35S_-GFP* at 48 and 72 hr after agro-infiltration. In contrast, there was no significant difference in the level of GFP accumulated in leaf tissues expressing each type of constructs (Figure 4). This results indicates that greater RCY1 accumulation in *N. benthamiana* leaves transiently expressing *P_RCY1_-gRCY1-HA::P_35S_-GFP* is not caused by differential efficiency of T-DNA transfer into the cells of the agro-infiltrated tissues, but by differences in the regulatory elements controlling genomic *RCY1* versus CaMV35S promoter-driven *RCY1* cDNA expression in *N. benthamiana*, just as in transgenic *Arabidopsis*.

### Comparison of RCY1 protein and *RCY1* transcript accumulation in *N. benthamiana* transiently expressing a series of native genomic *RCY1* and CaMV35S promoter-driven *RCY1* cDNA constructs

**Figure 5:**
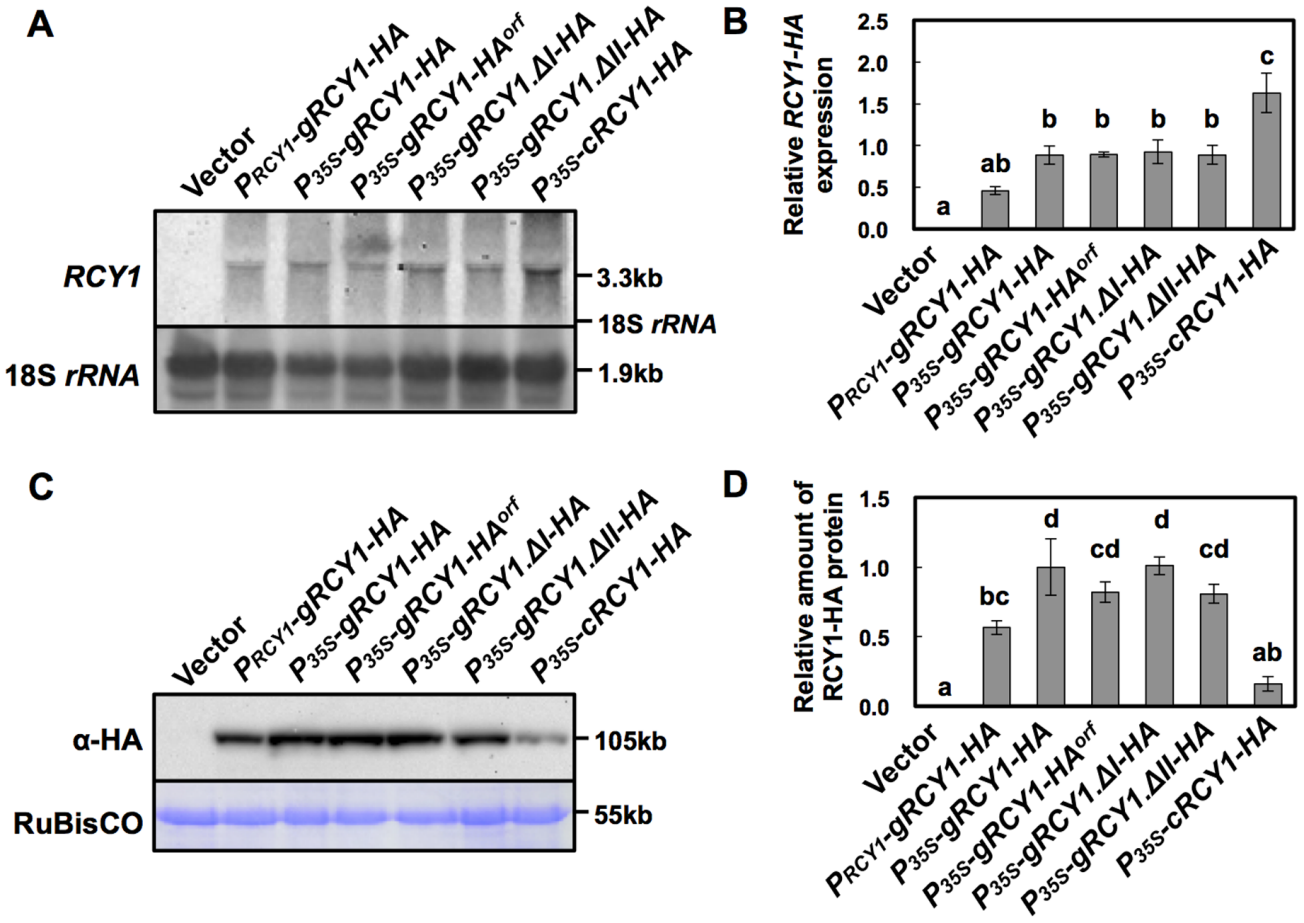
Detection of HA-epitope-tagged RCY1 protein and *RCY1* transcript in *N. benthamiana* leaves transiently expressing a series of *RCY1-HA* constructs under control of the *RCY1* or CaMV 35S promoters. *RCY1* transcripts in *N. benthamiana* leaves agro-infiltrated with *P_RCY1_-gRCY1-HA*, *P_35S_-gRCY1-HA*, *P_35S_-gRCY1-HA^orf^*, *P_35S_-gRCY1.ΔI-HA*, *P_35S_-gRCY1.ΔII-HA*, or *P_35S_-cRCY1-HA* were detected by northern hybridization. pRI201-AN (Vector) was used as an empty-vector control for agro-infiltration. As an internal control for RNA sample quantities, 18S *rRNA* is shown (A). Relative amounts of *RCY1* transcripts in each line were measured by quantitative RT-PCR. *EFα* gene expression was used as a standard for normalization of *RCY1* expression (B). HA-epitope-tagged RCY1 protein (α-HA) in *N. benthamiana* leaves transiently expressing *P_RCY1_-gRCY1-HA*, *P_35S_-gRCY1-HA*, *P_35S_-gRCY1-HA^orf^*, *P_35S_-gRCY1.ΔI-HA*, *P_35S_-gRCY1.ΔII-HA*, or *P_35S_-cRCY1-HA* was immunologically detected using anti-HA monoclonal antibody. As an internal control for protein sample quantities, the large subunit of RuBisCO was visualized by staining with CBB (C). RCY1-HA protein amounts in each line were quantified by band intensity using Quantity One software. For all experiments, four independent plants transiently expressing each vector construct were analyzed (D). The averages of relative *RCY1* transcript amounts ±SE are shown in B and D. In A and C, representative photographs are shown. The size of each band and the position of 18S *rRNA* were shown at right side of the panels. Data were subjected to analysis of variance and treatment means were compared by Tukey's test. Different letters indicate a statistically significant difference in the relative amount of *RCY1* transcript (*n* = 4, *P*<0.05).

To compare the activities of the native *RCY1* genomic and CaMV35S promoters for expressing the *RCY1*-coding region, *P_RCY1_-gRCY1-HA* and the construct *P_35S_-gRCY1-HA*, consisting of an HA-tagged genomic *RCY1*-coding region under the control of CaMV35S promoter (Figure 2) and including the 66-bp 5′-UTR and 71-bp 3′-UTR were each transiently expressed in *N. benthamiana*. As shown in Figure 5, the level of both *RCY1* transcript and RCY1 protein accumulation in leaf tissues expressing *P_35S_-gRCY1-HA* were significantly higher than those of *P_RCY1_-gRCY1-HA*, suggesting that the activity of the CaMV35S promoter is much stronger than that of the genomic *RCY1* promoter, just as we predicted. Therefore, increased RCY1 accumulation in leaf tissues of *A. thaliana* transformed with the genomic *RCY1* construct (Figure 3) or in *N. benthamiana* transiently expressing genomic *RCY1* (Figure 4) is not simply caused by the higher activity of the genomic *RCY1* promoter than the CaMV35S promoter.

Next, to analyze the roles of the 66-bp 5′-UTR and the 71-bp 3′-UTR of *RCY1* in *P_35S_-gRCY1-HA* in increased RCY1 accumulation, *P_35S_-gRCY1-HA^orf^* was constructed by deletion of the 5′- and 3′-UTRs from *P_35S_-gRCY1-HA* (Figure 2). When *P_35S_-gRCY1-HA* and *P_35S_-gRCY1-HA^orf^* were each agro-infiltrated into different regions of a single *N. benthamiana* leaf, *RCY1* transcript and RCY1 protein accumulated at similar levels between leaf tissues transiently expressing *P_35S_-gRCY1-HA* or *P_35S_-gRCY1-HA^orf^* (Figure 5). This result suggests that the 5′- and 3′-UTR regions of *RCY1* might not be associated with difference in RCY1 accumulation in *N. benthamiana* leaf tissues expressing genomic *RCY1*.

However, the accumulation of RCY1 in leaf tissues transiently expressing *P_35S_-gRCY1-HA* was much higher than that in leaf tissue expressing *P_35S_-cRCY1-HA* (Figure 5C and D). But the level of *RCY1* transcript in leaf tissues expressing *P_35S_-gRCY1-HA* was significantly lower than that in leaf tissues transiently expressing *P_35S_-cRCY1-HA* (Figure 5A and B). The vector *P_35S_-gRCY1-HA* contains the genomic intron-containing *RCY1* with all of its introns, whereas the vector *P_35S_-cRCY1-HA* contains the *RCY1* cDNA, which has no introns, both under the control of CaMV35S promoter (Figure 2). Together, these facts suggest that the existence of introns in *RCY1*-coding region could be indispensable for elevated level of RCY1 accumulation accompanied by decreased *RCY1* transcript level.

To further analyze the role of two introns in the *RCY1*-coding region for this intron-mediated enhancement (IME) of RCY1 accumulation, constructs with *RCY1* including either first or second intron in its coding region, *P_35S_-gRCY1.ΔI-HA* and *P_35S_-gRCY1.ΔII-HA* (Figure 2), were each transiently expressed in *N. benthamiana* leaves under control of the CaMV35S promoter. The level of RCY1 accumulation in each leaf tissue agro-infiltrated with *P_35S_-gRCY1.ΔI-HA* or *P_35S_-gRCY1.ΔII-HA* was similar to that in tissues expressing *P_35S_-gRCY1-HA* but was significantly higher than that in tissues expressing *P_35S_-cRCY1-HA* (Figure 5C and D). However, the transcript abundance of *RCY1* in leaf tissue agro-infiltrated with *P_35S_-gRCY1.ΔI-HA* or *P_35S_-gRCY1.ΔII-HA* was the same as that of tissues expressing *P_35S_-gRCY1-HA*, but much lower than that of *P_35S_-cRCY1-HA* transcript (Figure 5 A and B). Thus, both introns of genomic *RCY1* seem to be equally important for IME of RCY1 accumulation.

As shown in Figure 5, the level of full-length *RCY1* mRNA was inversely related to the accumulation of RCY1 in the leaf tissues transiently expressing either the intron-containing genomic *RCY1* or those expressing the intron-less *RCY1* cDNA. For some other *R* genes, alternative transcripts are required to induce complete resistance against pathogens [34]. However, analysis of *RCY1* transcript by northern hybridization using DIG-labeled cDNA probes corresponding to the sequence coding the LRR domain of RCY1 suggests that no alternative *RCY1* transcript but the full-length *RCY1* mRNA was observed in *N. benthamiana* leaf tissues transiently expressing either *P_35S_-gRCY1-HA* or *P_35S_-cRCY1-HA* (Figure S1).

### Acceleration of the defense reaction in *N. benthamiana* transiently expressing genomic *RCY1*

**Figure 6:**
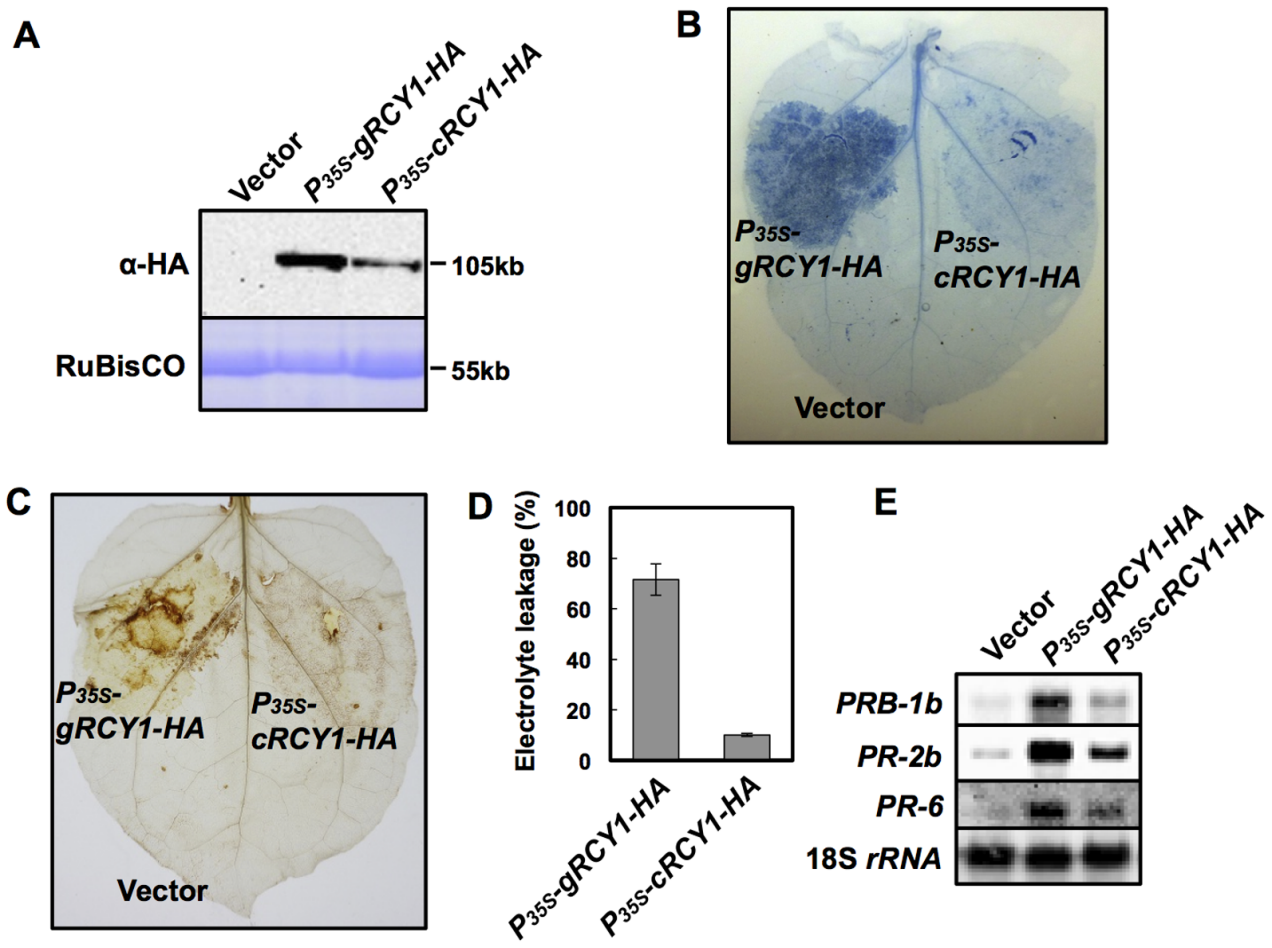
Activation of defense reaction in *N. benthamiana* leaves transiently expressing *P_35S_-gRCY1-HA* and *P_35S_-cRCY1-HA* under control of the CaMV 35S promoter. HA-epitope-tagged RCY1 protein (α-HA) (A) in *N. benthamiana* leaves transiently expressing *P_35S_-gRCY1-HA*, *P_35S_-cRCY1-HA*, or pRI201-AN (Vector) as an empty-vector control was immunologically detected using anti-HA monoclonal antibody. As an internal control for protein sample quantities, the large subunit of RuBisCO was visualized by staining with CBB. The size of each band was shown at right side of the panel. In *N. benthamiana* leaves transiently expressing *P_35S_-gRCY1-HA* or *P_35S_-cRCY1-HA*, hypersensitive response (HR) cell death was visualized by trypan blue staining (B), and H_2_O_2_ production was detected by DAB staining (C). To evaluate HR-cell death quantitatively, electrolyte leakage (D) in *N. benthamiana* leaves transiently expressing *P_35S_-gRCY1-HA*, *P_35S_-cRCY1-HA*, or empty-vector control was measured. Expression of the defense-related genes *PRB-1b*, *PR-2b*, and *PR-6* in *N. benthamiana* leaf tissue transiently expressing *P_35S_-gRCY1-HA*, *P_35S_-cRCY1-HA*, or the empty-vector control was analyzed by northern hybridization (E). As an internal control for RNA sample quantities, 18S *rRNA* was shown.

In *N. benthamiana* leaf tissues transiently accumulating RCY1, HR-like cell death is spontaneously induced [24]. Pathogen-independent autoactivation of defense reaction triggered by overexpression of some other *R*-gene has been reported [22]–[24]. HR-like cell death developed in *N. benthamiana* leaf tissues accumulating RCY1 can be considered as autoactivation of defense reaction [24]. When different regions of one fully expanded *N. benthamiana* leaf were infiltrated with *P_35S_-gRCY1-HA* or *P_35S_-cRCY1-HA*, leaf tissues expressing *P_35S_-gRCY1-HA*, which accumulated increased levels of RCY1-HA exhibited a greater magnitude of HR-like cell death than did leaf tissues transiently expressing *P_35S_-cRCY1-HA*, which accumulated relatively lower amounts of RCY1-HA (Figure 6A and B). The levels of electrolyte leakage and H_2_O_2_ production also increased in leaf tissues transiently expressing *P_35S_-gRCY1-HA* in comparison with *P_35S_-cRCY1-HA* (Figure 6C and D). Furthermore, the expression levels of several defense-related genes including *Pathogenesis-Related 1b* (*PRB-1b*), *PR-2b* and *PR-6* were much more induced in leaf tissues expressing *P_35S_-gRCY1-HA* than in those expressing *P_35S_-cRCY1-HA* (Figure 6E). Thus, intron-mediated enhancement (IME) of RCY1 accumulation seems to result in the acceleration of the defense reaction.

### Ability of each intron to increase RCY1 accumulation is not sequence specific

We constructed *RCY1intI+II-P_35S_-GUS* by inserting the DNA fragment containing the first and second introns of *RCY1* upstream of the CaMV35S promoter fused to theβ-glucronidase-coding region (*P_35S_-GUS*) (Figure S2A). This construct was used to analyze the possible function of the introns in genomic *RCY1* for IME of RCY1 accumulation. When *P_35S_-GUS* and *RCY1intI+II-P_35S_-GUS* were transiently expressed in different tissues within a single *N. benthamiana* leaf, the presence of neither *RCY1* introns upstream of the promoter affected the accumulation of RCY1 (Figure S2B). Therefore, these two *RCY1* introns may not function in IME of RCY1 accumulation through activation of promoter-mediated transcriptional process.

In general, the introns seem to be required to drive the correct expression pattern of the endogenous genes [39]. Some introns are known to be required to regulate gene expression at high level, while others have no effect or negative effects on gene expression [40]. To determine whether the ability of intron to increase RCY1 accumulation is specific to these *RCY1* introns, the native *RCY1* introns were replaced by other introns with no sequence homology to *RCY1* introns. The insertion of the intron of either *COLD-REGULATED 15A* (*COR15a*) or *PROFILIN 3* (*PRF3*) into the position near the transcriptional starting site of *GUS* reporter construct does not enhance *GUS* expression in *A. thaliana* [40], [41]. Thus, we first confirmed that the introns of *COR15a* and *PRF3* had no effect on the expression of those genes. Indeed, as shown with Figure S3 and S4, we compared *COR15a* transcript levels and COR15a accumulation between leaf tissue transiently expressing HA epitope-tagged *COR15a* with or without introns (*P_35S_-gCOR15-HA* or *P_35S_-cCOR15-HA*) under the control of CaMV35S promoter in *N. benthamiana* leaves. Our results suggested that neither *COR15a* transcript levels nor COR15a accumulation differed depending on the presence or absence of these introns (Figure S4A, C and E). Furthermore, when HA-tagged *PRF3* constructs (*P_35S_-gPRF3-HA* and *P_35S_-cPRF3-HA*) with or without introns were transiently expressed in *N. benthamiana* leaf tissues, the accumulation of *PRF3* transcripts and PRF3 protein were also unaffected (Figure S4B, D and E). These results suggest that the *COR15a* or *PRF3* introns do not function in IME of expression in either *A. thaliana* or *N. benthamiana*.

**Figure 7:**
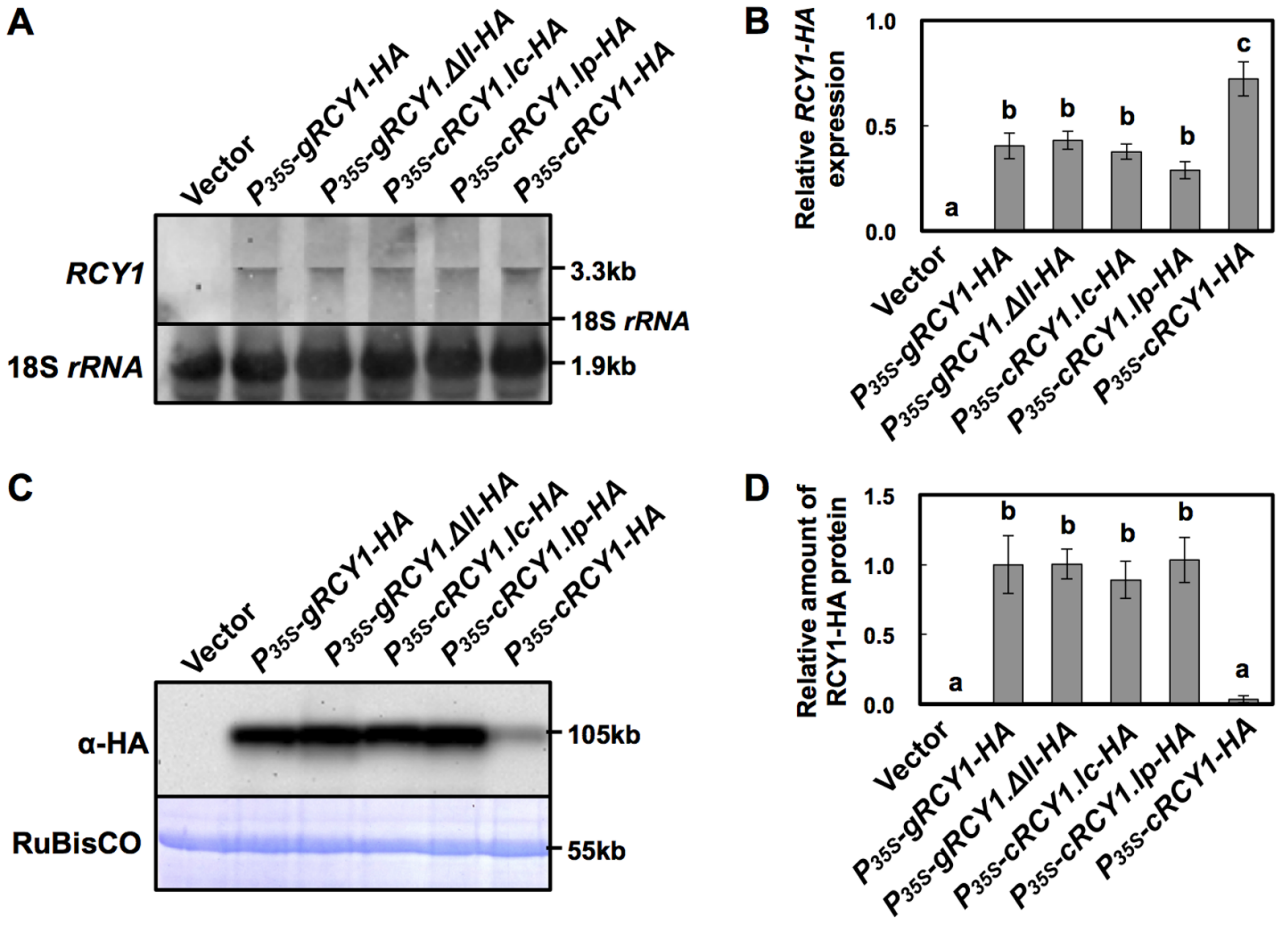
Detection of HA-epitope-tagged RCY1 protein and *RCY1* transcript in *N. benthamiana* leaves transiently expressing *RCY1-HA* constructs in which the *RCY1* introns were replaced with *COR15a* or *PRF3* introns. *RCY1* transcripts in *N. benthamiana* leaves agro-infiltrated with *P_35S_-gRCY1-HA*, *P_35S_-gRCY1.ΔII-HA*, *P_35S_-cRCY1.Ic-HA*, *P_35S_-cRCY1.Ip-HA*, or *P_35S_-cRCY1-HA* were detected by northern hybridization. pRI201-AN (Vector) was used as an empty-vector control for agro-infiltration. As an internal control for RNA sample quantities, 18S *rRNA* is shown (A). Relative amounts of *RCY1* transcripts in each line were measured by quantitative RT-PCR (B). HA-epitope-tagged RCY1 protein (α-HA) in *N. benthamiana* leaves transiently expressing *P_35S_-gRCY1-HA*, *P_35S_-gRCY1.ΔII-HA*, *P_35S_-cRCY1.Ic-H*A, *P_35S_-cRCY1.Ip-HA*, or *P_35S_-cRCY1-HA* was immunologically detected using anti-HA monoclonal antibody. As an internal control for protein sample quantities, the large subunit of RuBisCO was visualized by staining with CBB (C). RCY1-HA protein amounts in each line were quantified by band intensity using Quantity One software (D). For all experiments, four independent plants transiently expressing each vector construct were analyzed. The averages of relative amounts of *RCY1* transcript and protein ±SE are shown in B and D, respectively. In A and C, representative photographs are shown. The size of each band and the position of 18S *rRNA* were shown at right side of the panels. Data were subjected to analysis of variance and treatment means were compared by Tukey's test. Different letters indicate a statistically significant difference in the relative amount of *RCY1* transcript (*n* = 4, *P*<0.05).

Subsequently, the *COR15a* second intron or the *PRF3* first intron was integrated into the first exon junction position of *P_35S_-cRCY1-HA*, and the resulting constructs were designated *P_35S_-cRCY1.Ic-HA* and *P_35S_-cRCY1.Ip-HA*, respectively (Figure 2). The accumulation of RCY1-HA in leaf tissue transiently expressing *P_35S_-cRCY1.Ic-HA* or *P_35S_-cRCY1.Ip-HA* was very similar to that in leaf tissue expressing *P_35S_-gRCY1-HA* containing introns I and II or *P_35S_-gRCY1.ΔII-HA* containing intron I, but was much higher than that in leaf tissues expressing *P_35S_-cRCY1-HA* (Figure 7C and D). In contrast, *RCY1* transcript levels were also similar among leaf tissues transiently expressing intron-containing *RCY1-HA* constructs (*P_35S_-gRCY1-HA*, *P_35S_-gRCY1.ΔII-HA*, *P_35S_-cRCY1.Ic-HA* and *P_35S_-cRCY1.Ip-HA*), but were much lower than in leaf expressing intron-less *RCY1-HA* (*P_35S_-cRCY1-HA*) (Figure 7A and B). These results indicate that IME of RCY1 accumulation does not dependent on the specific sequence of *RCY1* introns, which are exchangeable for other introns from other genes having no IME activity.

## Discussion

Gene expression is tightly regulated through a combination of transcriptional and post-transcriptional control mechanisms. Aside from epigenetic factors, many elements of a gene's sequence structure, including the promoter, enhancers, the introns, and the 5′ and 3′-UTRs contribute to the expression level of a given gene at any point in time [39]. In this study, the analysis of RCY1 accumulation in *N. benthamiana* leaves transiently expressing a series of *RCY1* constructs reveals that the presence of the intron in the *RCY1* coding region seems to be indispensable to achieve an appropriate level of RCY1 expression to provide complete resistance to CMV(Y), while the effect of the intron on gene expression is often eclipsed by the function of other elements. To our knowledge, this is the first report indicating that an intron in the coding region of an *R* gene is directly associated with the level of R-protein accumulation. The enhancement of the *RCY1*-mediated defense reaction via elevated RCY1 protein accumulation appears to indicate that maintenance of appropriate R-protein levels in host plants is important for suppression of virus multiplication, restriction of the spread of virus around the primary virus infection site [21], [24], and induction of the defense reaction (Figure 6). Therefore, the intron-mediated enhancement of RCY1 expression seems to play a key role in complete resistance to CMV(Y) in *Arabidopsis*.

Introns do seem to be required to drive correct expression patterns in diverse organisms including plants. The positive effect of introns on gene expression has been named intron-mediated enhancement (IME) [40], [42]. IME of gene expression in plants is generally associated with up-regulation of mRNA levels, which often increases the accumulation of the corresponding gene product. Actually, many plant gene introns have been shown to induce gene expression at the transcriptional level [40], [43]–[47]. However, it is still unclear exactly how the presence of an intron in a gene affects the level of gene expression. Splicing of introns may induce the modification of transcripts, such as capping or polyadenylation to increase transcript stability. IME signals, predicted by the IMEter algorithm for estimating the efficiency of IME in *A. thaliana*, have recently been reported to be most abundant in introns located in the 5′-UTRs and coding regions near the transcription start site [40], [48]. But remarkably, for IME of *RCY1*, RCY1 protein accumulation was not preceded by increases in levels of *RCY1* transcript, but by relatively decreased transcript levels in *A. thaliana* and *N. benthamiana* (Figures 3 and 5). *GUS* expression was not enhanced by insertion of either of two *RCY1* introns upstream of the CaMV 35S promoter (Figure S2). However, IME of RCY1 accumulation was observed even when the introns of *RCY1* were replaced with *COR15a* or *PRF3* introns that lack IME signals (Figure 7) [40]. These facts suggest that IME of RCY1 accumulation does not operate by either a typical transcriptional enhancer in the introns or by an increase in the steady state quantity of mature mRNA. The mechanism here may differ from IME observed to date, which is typified by an increase in mRNA accumulation controlled by IME signal-containing introns located near the 5′-end of a gene.

Alternative transcripts have been detected in a certain number of known NB-LRR-encoding *R* genes [17], [25]–[32]. However, the direct evidence of the requirement of alternative splicing for *R*-gene-mediated complete resistance is just limited in some well-characterized *R*-genes: e.g. *N* and *RPP4* [29], [33], thus I think that the role of alternative splicing in *R*-gene-mediated disease resistance is still unclear. Intron retention is a major event in alternative splicing. However, since any alternative *RCY1* transcript was not detected by northern blot analysis (Figure S1), we speculate less possibility that alternative splicing occurs in *RCY1* gene expression. Although it seems to be not easy to distinguish alternative transcript from incompletely spliced heteronuclear RNA by RT-PCR, it is necessary to further determine whether splice variants reside with ribosomes in cytoplasm and are required for *RCY1*-conferred resistance to CMV(Y).

Following intron splicing, the exon junction complex (EJC), a protein complex that localizes near the junction of two exons (20–25 nucleotides upstream), is deposited on the mRNA, and functions from intracellular transport of mRNA through translation of mRNA [49]. In animals, the EJC, which contains several proteins including core components Y14, Mago, eIF4AIII, and MLN51, is required for increased translation of spliced mRNA via interaction between Y14, Mago, and PYM (Partner of Y14 and mago), which then associate with the 40S ribosomal subunit in the cytoplasm [49], [50]. In plants, there is insufficient evidence to suggest that EJC plays an important role in the translation of mRNA. However, homologues of Y14, Mago, and PYM have been identified in *A. thaliana* and some other plant species [51]–[53]. Therefore, a translational control system mediated by EJC with its interactor PYM might function in IME of RCY1 accumulation in *A. thaliana* and *N. benthamiana*. In next step, it is necessary to confirm the physical interaction of EJC with the region near the junction of the exon of *RCY1* mRNA or identify other host factors interacting with the exon junction sequence.

In *A. thaliana*, 118 out of 149 NB-LRR-encoding genes and 7 out of 58 related genes contain variable numbers of introns, whereas the remaining 31 NB-LRR-encoding genes and 51 related genes contain no introns [6]. Therefore, the role of introns is under focus for understanding the regulatory mechanisms of NB-LRR-class *R*-gene expression. Molecular dissection of the machinery for regulating intron-mediated *RCY1* expression will provide a new platform for the design of gene structures that are more effective than simple overexpression of *R* genes for regulating *R*-gene expression and thereby conferring complete resistance to pathogens.

## Materials and Methods

### Plant and Virus

A yellow strain of *Cucumber mosaic virus* [CMV(Y)] [54] was propagated on *N. tabacum* ‘Xanthi nc’, and the virus was purified as previously described [55]. *Nicotiana benthamiana* was grown on conventional soil in a growth chamber (KG-50HLA; Koito Manufacturing Co. Ltd., Tokyo) at day and night temperatures of 25°C and 22°C, respectively, under a 14 h photoperiod at 10,000 lux. *A. thaliana* ecotype Columbia (Col-0): three independent lines of Col::pRCY1-HA [21] #8, #10 and #12; and three independent lines of Col::P_35S_-cRCY1-HA: #5, #21 and #23 which were generated in this study, were grown on soilless mix (PRO-MIX B; Premier Horticulture, Ltd., Quakertown, PA, USA) at 25°C under continuous illumination (8,000 lux).

### Vector construction

Ten vector constructs encoding HA-tagged RCY1 are shown in Figure 2. For all cloning manipulations, the In-Fusion HD Cloning System (Clontech-TAKARA, Kyoto, Japan) was used according to the manufacturer's instruction manual. To generate *P_RCY1_-gRCY1-HA*, a genomic DNA fragment of *RCY1* including the promoter region from approximately 1.5 kb upstream of the start codon and the 71-bp 3′ sequence downstream of the *RCY1* stop codon (71-bp 3′-UTR) was amplified by PCR. For this reaction, the primers RI201AN.HindIII15.gRCY1 and RI201AN.SalI15.RCY1utr3 (Table S1) were used with pBS+SK/RCY1-HA [21] as a template, in which a HA-epitope tag sequence was inserted at the 3′-end of the RCY1-coding region. After purification of the PCR product according to the standard protocol [56], the purified DNA fragment was cloned upstream of the *Heat Shock Protein* gene (T_HSP_) terminator in pRI201-AN (Takara-BIO, Mie, Japan) that had been linearized by *Hin*dIII and *Sal*I digestion. pRI201::RCY1-HA which we constructed previously [24] was renamed *P_35S_-cRCY1-HA* in this study.

To generate modified *P_35S_-cRCY1-HA* and *P_RCY1_-gRCY1-HA* vectors carrying the CaMV35S promoter (P_35S_)-GFP coding region (GFP)-T_NOS_ as an internal control, a P_35S_-GFP-T_NOS_ fragment was amplified by PCR using the primers pUC.-40+NotI.15R and pUC.RV+NotI.15F (Table S1) with the *35Spro:GFP* construct [57] as a template, and then purified according to the standard protocol [56]. The purified P_35S_-GFP-T_NOS_ fragment was cloned into the *Not*I site of *P_35S_-cRCY1-HA* and *P_RCY1_-gRCY1-HA*. The resulting vector constructs were designated *P_35S_-cRCY1-HA*::*P_35S_-GFP* and *P_RCY1_-gRCY1-HA*::*P_35S_-GFP*, respectively.

To construct *P_35S_-gRCY1-HA*, a DNA fragment containing the RCY1-coding genomic region tagged with an HA-epitope sequence at its 3′-end, and including both the 66-bp 5′ sequence upstream of the *RCY1* start codon (66-bp 5′-UTR), and the 71-bp 3′-UTR, was amplified by PCR with the primers RI201AN.NdeI15.RCY1utr5 and RI201AN.SalI15.RCY1utr3 (Table S1) using pGA482/RCY1-HA [21] as a template. After purification by the standard protocol [56], the PCR product was inserted between the CaMV 35S promoter (P_35S_) fused to the 5′-UTR sequence of *Arabidopsis Alcohol Dehydrogenase* gene (ADH5′-UTR) and the T_HSP_ in pRI201-AN that had been linearized with *Nde*I and *Sal*I. To create the *P_35S_-gRCY1-HA^orf^* construct containing the HA-tagged *RCY1* coding region under the control of CaMV 35S promoter but without it 66-bp 5′-UTR and 71-bp 3′-UTR, the PCR product amplified using the primers RI201AN.NdeI15bp and RI201AN.SalI15bp (Table S1) with pGA482/RCY1-HA [21] as a template was cloned downstream of P_35S_ between the ADH5′-UTR and the T_HSP_ terminator of *Nde*I- and *Sal*I-digested pRI201-AN.

*P_35S_-gRCY1.ΔI-HA* and *P_35S_-gRCY1.ΔII-HA* were derived from *P_35S_-gRCY1-HA*, but lack either the first or second intron of the RCY1-coding region, respectively. The inserts for each of these constructs were generated by fusing two other PCR fragments in PCR. For *P_35S_-gRCY1.ΔI-HA*, a PCR fragment containing the 5′ half of the cDNA-derived RCY1-coding sequence with no introns was amplified with the primers RI201AN.NdeI15.RCY1utr5 and RCY1.ExonII-R (Table S1) using *P_35S_-cRCY1-HA* as a template. The second PCR fragment containing the 3′ half of the RCY1-coding sequence including the second intron was amplified by PCR with the primers RCY1.ExonII-F (Table S1) and RI201AN.SalI15.RCY1utr3 using *P_35S_-gRCY1-HA* as a template. The final PCR fragment was amplified with the primers RI201AN.NdeI15.RCY1utr5 and RI201AN.SalI15.RCY1utr3 using a mixture of the first and second PCR fragments above as templates, was purified, then was cloned downstream of P_35S_ between the ADH5′-UTR and T_HSP_ terminator in pRI201-AN linearized by *Nde*I and *Sal*I-digestion. To generate *P_35S_-gRCY1.ΔII-HA* lacking the second *RCY1* intron, two PCR fragments were amplified with two different sets of primers: RI201AN.NdeI15.RCY1utr5 and RCY1.ExonII-R using *P_35S_-gRCY1-HA* as a template; and RCY1.ExonII-F and RI201AN.SalI15.RCY1utr3 using *P_35S_-cRCY1-HA* as a template, respectively. A mixture of these two PCR products was used as a template for final PCR with the primers RI201AN.NdeI15.RCY1utr5 and RI201AN.SalI15.RCY1utr3. The resulting PCR product was purified and cloned downstream of P_35S_ between the ADH5′-UTR and the T_HSP_ terminator in pRI201-AN.

Introns of Arabidopsis *COLD-REGULATED 15A* (*COR15a*, At2g42540) [58] and *PROFILIN 3* (*PRF3*, At5g56600) [41] do not have the activity of intron-mediated enhancement for their expression [41], [40]. The HA-epitope-tagged sequence of *COR15a* including its intron was amplified by PCR with the primers RI201AN.NdeI.COR15a-F and RI201AN.SalI.V.COR15a-R (Table S1) for *COR15a* using genomic DNA extracted from *A. thaliana* ecotype Col-0 as template. After purification, this fragment was cloned into the *Nde*I and *Sal*I sites of pRI201-AN, and the resulting construct was named *P_35S_-gCOR15a-HA* (Figure S3). The HA-epitope-tagged sequence of *PRF3* including its introns was amplified by PCR with the primers RI201AN.NdeI.PRF3-F and HA.BstZ17I.PRF3-R (Table S1) using *A. thaliana* ecotype Col-0 genomic DNA as template. The purified PCR product was then cloned into the *Nde*I and *Bst*Z17I sites of *P_35S_-gCOR15a-HA* (Figure S3), and the resulting construct was designated *P_35S_-gPRF3-HA*. The cDNAs of *COR15a* and *PRF3* without introns were amplified by PCR with the same primers as immediately above using first-strand cDNA as a template, which had been reverse-transcribed from total RNA extracted from cold-assimilated Col-0. These purified PCR products were then cloned into either the *Nde*I and *Sal*I sites of pRI201-AN or the *Nde*I and *Bst*Z17I sites of *P_35S_-gCOR15a-HA*, respectively. The resulting constructs were designated as *P_35S_-cCOR15a-HA* and *P_35S_-cPRF3-HA*, respectively (Figure S3).

An RCY1-coding DNA fragment containing the *COR15a* intron at its first splice junction site, was constructed by fusing three purified DNA fragments individually amplified by PCR separate reactions with the following three sets of primers: RI201AN.NdeI15.RCY1utr5 and nE1rcy.cIcor-R (Table S1) for the first exon of *RCY1*; nE1rcy.cIcor-F and nIcor.cE2rcy-R (Table S1) for the *COR15a* second intron; and nIcor.cE2rcy-F (Table S1) and RI201AN.SalI15.RCY1utr3 for second exon of *RCY1*. The reactions to produce the above three fragments were then followed by second PCR with a new set of primers: RI201AN.NdeI15.RCY1utr5 and RI201AN.SalI15.RCY1utr3, using the purified PCR products above as a template.

The RCY1-coding DNA fragment with the *PRF3* intron added at its first splicing junction site was also used as template for amplification in three individual PCR with the following three sets of primers: RI201AN.NdeI15.RCY1utr5 and nE1rcy.cIprf3-R (Table S1) for the first exon of *RCY1*; nE1rcy.cIprf3-F and nIprf3.cE2rcy-R (Table S1) for the *PRF3a* first intron; and nIprf3.cR2rcy-F (Table S1) and RI201AN.SalI15.RCY1utr3 for the second exon of *RCY1* (Table S1). The reactions to produce the above three fragments were then followed by a second PCR with the primers RI201AN.NdeI15.RCY1utr5 and RI201AN.SalI15.RCY1utr3 using the purified PCR products immediately above as a template. The two resulting purified DNA fragments were then cloned into the *Nde*I and *Sal*I sites of pRI201-AN, respectively, and the resulting constructs were designated as *P_35S_-cRCY1.Ic-HA* and *P_35S_-cRCY1.Ip-HA* (Figure 2).

Genomic DNA to be used as PCR template was extracted from the leaves of *A. thaliana* ecotype Col-0 by the CTAB method [59]. Total RNA to be used as template for reverse-transcriptional PCR was extracted from Col-0 leaves using an RNeasy Plant Mini Kit (Qiagen, Hilden, Germany) according to the manufacturer's instructions. First-strand RNA was reverse transcribed from total RNA using Thermoscript RT-PCR System (Invitrogen, Carlsbad, California, USA) containing oligo(dT)_20_ primer according to the manufacturer's instruction manual.

All desired constructs were confirmed by restriction patterns and the Sanger sequencing method using a CEQ 8000 Automated DNA Sequencer (Beckman Coulter, Brea, CA, USA). Each plasmid (100 ng) was introduced into *Agrobacterium tumefaciens* LBA4404 (Takara-BIO) by electroporation according to the standard protocol [56].

### Transformation of *A. thaliana*

*A. thaliana* ecotype Col-0 plants were transformed with *Agrobacterium tumefaciens* LBA4404 containing *P_35S_-cRCY1-HA* by vacuum infiltration [60]. Transgenic plants were screened on 0.5× MS medium [61] with 0.8% agar and 50 µg of kanamycin per ml. Transformation of *P_35S_-cRCY1-HA* into Col-0 plants was confirmed in second and third generation (T2 and T3) plants by rat anti-HA monoclonal antibodies (clone 3F10, dilution 1∶10,000; Roche, Indianapolis, IN, USA), according to the method described previously [21]. These transgenic plants were designated as line Col::P_35S_-cRCY1-HA. The *RCY1* transgene was detected by quantitative PCR using the 7300 Real-Time PCR System (Applied Biosystems, Foster City, CA, USA) with the primers RCY1-HA.F and RCY1-HA.R (Table S2) and genomic DNA extracted from each transformant as a template.

### Transient expression of *RCY1* constructs in *N. benthamiana*

*Agrobacterium*-mediated transient expression of the following ten vector constructs: *P_35S_-cRCY1-HA*, *P_RCY1_-gRCY1-HA, P_35S_-cRCY1-HA::P_35S_-GFP*, *P_RCY1_-gRCY1-HA::P_35S_-GFP*, *P_35S_-gRCY1-HA*, *P_35S_-gRCY1-HA^orf^*, *P_35S_-gRCY1.ΔI-HA*, *P_35S_-gRCY1.ΔII-HA*, *P_35S_-cRCY1.Ic-HA*, and *P_35S_-cRCY1.Ip-HA* (Figure 2) in N. benthamiana leaves was performed by the method described previously [24]. To compare levels of RCY1-HA accumulation among leaves expressing a set of vector constructs, independent regions of single fully expanded of N. benthamiana leaves were infiltrated with each Agrobacterium suspension containing each vector construct. RCY1-HA protein was detected by immunoblotting with rat anti-HA monoclonal antibody (clone 3F10, dilution 1∶10,000; Roche, Indianapolis, IN, USA) according to the method described previously [21]. The intensity of bands visualized with the ECL Prime chemiluminescent substrate (GE Healthcare, Piscataway, NJ, USA) was measured by Quantity One software using a VersaDoc MP 4000 system (Bio-Rad, Hercules, CA, USA). Four independent protein samples per experiment were used to quantitatively analyze HA-tagged protein levels, and the average band intensities ±SE are shown. GFP accumulation was immunologically detected by the method described previously [62] using antiserum against GFP (Medical and Biological Laboratories: MBL, Nagoya, Japan). As an internal control, the large subunit of RuBisCO was detected by staining with Coomassie Brilliant Blue R-250 (CBB) according to the standard protocol.

### Northern blot and quantitative RT-PCR

Total RNA was extracted from the leaves of *A. thaliana* and *N. benthamiana*, using an RNeasy Plant Mini Kit (Qiagen, Hilden, Germany) according to the manufacturer's instruction. Transcripts of the *RCY1* transgene and the *PR-1a* gene of *A. thaliana* were detected by northern hybridization according to the procedure described previously [37]. DIG-labeled probe for *Arabidopsis PR-1a* was prepared according to the procedure described previously [63]. DIG-labeled probes specific to the *RCY1* transgene in *A. thaliana* were amplified by PCR with the primers RCY1utr3-F and RCY1utr3-R (Table S3). To detect the transcripts in *N. benthamiana*, DIG-labeled probes for the *RCY1* transgene, and *PRB-1b*, *PR-2b*, and *PR-6* from *N. benthamiana* were amplified by PCR with the following sets of primers: RCY1.ExonI-F and rpp8-R3 for the *RCY1* transgene; PRB-1b-F and PRB-1b-R for *PRB-1b*; PR-2b-F and PR-2b-R for *PR-2b*; and PR-6-F and PR-6-R for *PR-6* (Table S3). Transcripts of *COR15a* and *PRF3* were detected with DIG-labeled probes amplified by PCR with the primers COR15a-F and COR15a-R for *COR15a* (Table S3); or PRF3-F and PRF3-R for *PRF3* (Table S3), respectively. All DNA probes were labeled with digoxigenin (DIG)-11-dUTP by PCR using a PCR DIG Synthesis Kit (Roche, Penzberg, Germany) according to the manufacturer's instructions. DIG-labeled probes were detected using an alkaline phosphatase-conjugated anti-DIG antibody and were visualized with the CDP-Star Reagent (New England Biolabs, Beverly, MA, USA) according to the instruction manuals.

For quantitative measurement of *RCY1* transgene transcripts, and the controls *UBQ5* of *A. thaliana*, and *EF-1a* in *N. benthamiana*, 1 µg total RNA was reverse transcribed into cDNA using the PrimeScript RT Reagent Kit with gDNA Eraser (Takara-Bio) containing random hexamer primers according to the manufacturer's instruction manual. Quantitative RT-PCR amplification for detecting *RCY1* transcripts was performed in triplicate 20 µl reactions containing template cDNA (2 µl), 0.4 µM *RCY1*-specific primers RCY1-HA.F and RCY1-HA.R (Table S2), 1xROX Reference Dye, and 1xSYBR *Premix Ex Taq* II (Tli RNase H plus) (Takara-Bio) using the 7300 Real-Time PCR System (Applied Biosystems). PCR conditions and data analysis were performed according to the procedure described previously [64]. As a standard control, the level of the *A. thaliana UBQ5* transcript or the *N. benthamiana EF1a* transcript was quantified by RT-PCR using SYBR with the primers RTUBQ5-F1 and RTUBQ5-R1 for *UBQ5* (Table S2) or NbEF1a-F and NbEF1a-R for *EF-1a* (Table S2). *RCY1* mRNA quantities were normalized relative to the values of constitutively expressed *UBQ5* or *EF-1a* mRNAs. In each experiment, three independent *A. thaliana* and four independent *N. benthamiana* plants were used for quantitative measurement of each transcript, and the level of gene expression is shown as the average ± SE of the value of *RCY1* mRNA relative to *UBQ5* or *EF-1a* mRNA. Data were subjected to analysis of variance and treatment means were compared by Tukey's test.

### Detection of cell death and H_2_O_2_ production

The development of HR cell death at 72 h after agro-infiltration with *RCY1* constructs was visualized by trypan blue staining according to a standard protocol [65]. To evaluate HR cell death quantitatively, electrolyte leakage was measured a method modified from that described by Kim et al. (2003) [66]. At 80 h after agro-infiltration with *RCY1* constructs, ten leaf discs 0.6 mm in diameter were floated on 11 ml of 0.4 M sorbitol and incubated at 25°C. After 20 h of incubation, the incubation solution was removed from the leaf discs, and the conductivity of the incubation solution was measured as sample conductivity with a conductivity meter (ES-51, HORIBA, Ltd., Kyoto, Japan). Ten leaf discs were then combined with the incubated solution and boiled for 10 min. The conductivity of the boiled solution was measured as total conductivity. Relative electrolyte leakage was expressed as the percentage of sample conductivity to total conductivity. Four independent plants were used for measurement of electrolyte leakage and the averages of relative amounts ±SE are shown.

H_2_O_2_ evolved due to HR cell death was detected by 3,3′-dianimobenzidine (DAB) staining. At 72 h after agro-infiltration with *RCY1* constructs, the infiltrated leaves were incubated with 1 mg/ml DAB solution for 12 h at 25°C in dark condition, and then washed in 95% ethanol at 94°C for 10 min.

### Analysis of transcriptional enhancer activity from introns of *RCY1*

A DNA fragment spanning the first intron to the second intron of *RCY1* genomic DNA was cloned upstream of the CaMV P_35S_ in pSMAHdN632L-M2GUS [67], which was then renamed *P_35S_-GUS* in this study. The intron fragment was amplified by PCR with the primers: SMAH.SbfIXhoI.RCYintron-F (5′-GTTAAGGAATTGCCCTGCA- GGCTCGAGTTCCACGGAAAAGAGGTG-3′, in which the 15-bp vector sequence for In-Fusion Cloning is underlined) and SMAH.SbfI.RCY1intron-R (5′-GGCTAATCT- GGGGACCTGCACATCAAGCCTTACTTCTGC-3′), using *P_35S_-gRCY1-HA* as a template. After purification of the PCR product according to the standard protocol [56], the fragment was cloned into pSMAHdN632L-M2GUS linearized by digestion with *Sbf*I, and the resulting vector was named *RCY1intI+II-P35S-GUS*.

GUS reporter accumulation was measured by ELISA. Leaf tissues transiently expressing each gene were homogenized in 100-fold volumes of 0.05 M Na_2_CO_3_. The protein concentration in the homogenate was determined by the Bradford method [68]. The homogenate was diluted 10-fold with 0.05 M Na_2_CO_3_ and subjected to ELISA according to the standard protocol [56] using a polyclonal anti-GUS antibody (Molecular Probes, Eugene, Oregon, USA). GUS accumulation is shown as absorbance at 405 nm per 0.22 mg/ml of total protein. Four independent leaf samples transiently expressing vector constructs were used for detection of GUS accumulation. To present quantitative measurement of GUS accumulation, the average absorbance ±SE values are shown.

## Supporting Information

Figure S1 **Detection of** ***RCY1*** **transcripts in** ***N. benthamiana*** **leaves transiently expressing** ***P_35S_-cRCY1-HA*** **and** ***P_35S_-gRCY1-HA*** **.** *RCY1* transcripts in *N. benthamiana* leaves agro-infiltrated with the intron-containing genomic *RCY1* coding region (*P_35S_-gRCY1-HA*), *RCY1* cDNA without introns (*P_35S_-cRCY1-HA*), or pRI201-AN (Vector) as an empty vector control were detected by northern hybridization. Full-length *RCY1* transcripts are indicated as bands marked by the arrow. RNA was extracted from three independent plants (1, 2, and 3) per vector-infiltrated plants.
(TIFF)

Figure S2 **Assay for the enhancement of promoter activity by the** ***RCY1*** **intron sequence.** Schematic of the β-glucronidase (*gusA*)-coding vector constructs: *P_35S_-GUS* with *gusA* under control of the CaMV 35S promoter (P_35S_) and *nopaline synthase* terminator (T_NOS_); *RCY1intI+II-P_35S_-GUS* in which *RCY1* intron sequences shown by the black fold lines, were inserted into *Sbf*I site upstream of P_35S_ of *P_35S_-GUS* (A). The position of insertion of the intron–containing fragment from *P_35S_-gRCY1-HA* into *RCY1intI+II-P_35S_-GUS* is indicated by dotted lines. Relative GUS protein quantities in *N. benthamiana* leaves transiently expressing *P_35S_-GUS* or *RCY1intI+II-P_35S_-GUS* were measured by ELISA at 0, 24, 48, and 72 h after agro-infiltration (B). Four independent plants transiently expressing each vector construct were analyzed. The averages of relative GUS protein amounts ±SE are shown in B.
(TIFF)

Figure S3 **Schematic structure of the** ***COR15a*** **and** ***PRF3*** **vector constructs under control of the CaMV 35S promoter.** *COR15a* or *PRF3* cDNA without introns but with HA-epitope tags (HA) at their 3′-ends were cloned between the CaMV 35S promoter with the 5′-UTR sequence of the *Arabidopsis Alcohol Dehydrogenase* gene (ADH5′-UTR) and *Heat Shock Protein* gene terminator (T_HSP_) in the pRI201-AN binary vector. The resulting constructs were named *P_35S_-cCOR15a-HA* and *P_35S_-cPRF3-HA*, respectively. The COR15a- or PRF3-coding regions are indicated by gray boxes and the two splice junction sites are indicated by vertical lines in the boxes. Genomic *COR15a* or *PRF3* tagged with HA at its 3′-end under control of the CaMV 35S promoter (*P_35S_-gCOR15a-HA* and *P_35S_-gPRF3-HA*) contains intron sequences indicated by the black dashed lines.
(TIFF)

Figure S4 **Comparison of HA-epitope-tagged COR15a and PRF3 transcript and protein levels among** ***N. benthamiana*** **leaf tissues transiently expressing intron-containing genomic** ***COR15a*** **or** ***PRF3*** **or** ***COR15a*** **or** ***PRF3*** **cDNAs without introns.** *COR15a* (A) or *PRF3* (B) transcripts in *N. benthamiana* leaf tissues transiently expressing either the intron-containing genomic *COR15a* (*P_35S_-gCOR15a-HA*) or *PRF3* (*P_35S_-gPRF3-HA*), or the cDNAs for *COR15a* (*P_35S_-cCOR15a-HA*) or *PRF3* (*P_35S_-cPRF3-HA*) without introns, were detected by northern hybridization. pRI201-AN (Vector) was used as an empty-vector control. As an internal control for RNA sample quantities, 18S *rRNA* is shown. The size of each band and the position of 18S *rRNA* were shown at right side of the panels. COR15a (C) or PRF3 (D) protein amounts in each line were quantified by band intensity using Quantity One software. Four independent plants transiently expressing each vector construct were analyzed. The averages of relative COR15a-HA and PRF3-HA protein amounts ±SE are shown. The COR15a-HA and PRF3-HA proteins in leaf tissues of each line were also detected by immunoblotting (E). As controls, pRI201-AN (*Vector*), *P_35S_-gRCY1-HA*, and *P_35S_-cRCY1-HA* were agro-infiltrated into *N. benthamiana* leaves. As an internal control for protein sample quantities, the large subunit of RuBisCO was visualized by staining with CBB. In this experiment, 1/50 volume of total protein sample of leaf accumulating COR15a-HA against that of RCY1-HA and PRF3-HA was applied on the gel, since the level of COR15a-HA accumulation was essentially much higher than others. The size of each band and the position of RuBisCO large subunit were shown at right side of the panel.
(TIFF)

Table S1 (XLSX)

Table S2 (XLSX)

Table S3 (XLSX)
