# Correction: Role of Intron-Mediated Enhancement on Accumulation of an *Arabidopsis* NB-LRR Class R*-*protein that Confers Resistance to *Cucumber mosaic virus*

**DOI:** 10.1371/journal.pone.0107185

**Published:** 2014-09-04

**Authors:** The PLOS ONE Staff

There are errors in Figures 3, 4, 5, 6, 7, and Figure S4 of the published article. In several places the units “kb” should read “kDa”. The corrected figures and their legends can be seen here.

**Figure 3:**
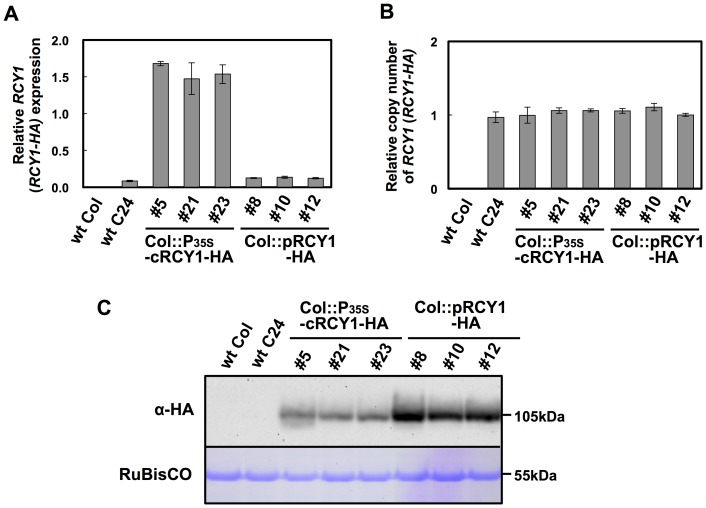
Detection of RCY1 protein, the *RCY1*transcript, and the *RCY1* transgene in three independent Col-0 lines transformed with HA-tagged genomic *RCY1* or HA-tagged *RCY1* cDNA without introns. Relative amounts of *RCY1* transcripts in wild-type *A. thaliana*ecotypes Col-0 (wt Col) and C24 (wt C24), and three independent lines transformed with HA-tagged genomic *RCY1* (Col::pRCY1-HA #8, #10, and #12) or HA-tagged *RCY1* cDNA without introns (Col::cRCY1-HA #5, #21 and #23), were measured by quantitative RT-PCR (A). Relative amounts of RCY1-coding transgene DNA in wild-type ecotypes (wt Col and wt C24) and three independent lines of Col::gRCY1-HA and Col::cRCY1-HA were measured by quantitative PCR using each genomic DNA as a template (B). HA-epitope-tagged RCY1 protein (α-HA) in wild-type ecotypes (wt Col and wt C24); Col::gRCY1-HA #8, #10 and #12 lines; and Col::cRCY1-HA #5, #21 and #23 lines was immunologically detected using monoclonal anti-HA epitope antibody. As an internal control for protein sample quantities, the large subunit of RuBisCO was visualized by staining by Coomassie Brilliant Blue R-250 (CBB) (C). For all experiments, three independent plants per vector were analyzed. The averages of relative *RCY1*transcript amounts ±SE are shown in A and B. In C, a representative photograph is shown. The size of each band was shown at right side of the panel.

**Figure 4:**
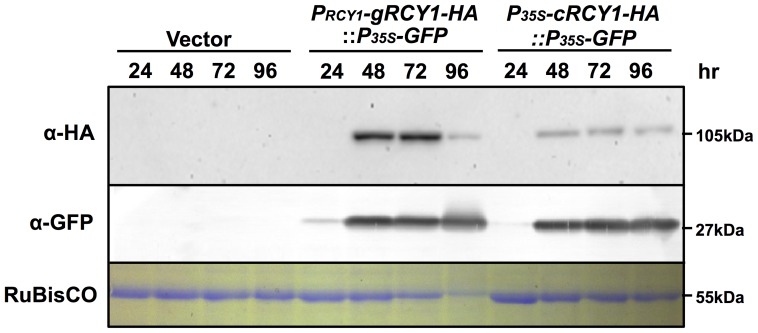
Detection of HA-epitope-tagged RCY1 in*N. benthamiana* leaves transiently expressing genomic *RCY1-HA* under control of the native *RCY1*promoter or the *RCY1* cDNA without introns under control of the CaMV 35S promoter. HA-epitope-tagged RCY1 protein (α-HA) in *N. benthamiana* leaf tissues transiently expressing *P_RCY1_-gRCY1-HA::P_35S_-GFP* or *P_35S_-gRCY1-HA::P_35S_-GFP* was immunologically detected using monoclonal antibody against the HA epitope at 24, 48, 72, and 96 h after agro-infiltration. GFP accumulation (α-GFP) was also immunologically detected at the same time points using polyclonal antibody against GFP as an internal standard. As an internal control for protein sample quantities, the large subunit of RuBisCO was visualized by staining with CBB. The size of each band was shown at right side of the panel. For all experiments, three independent plants per vector were analyzed and representative data are shown.

**Figure 5:**
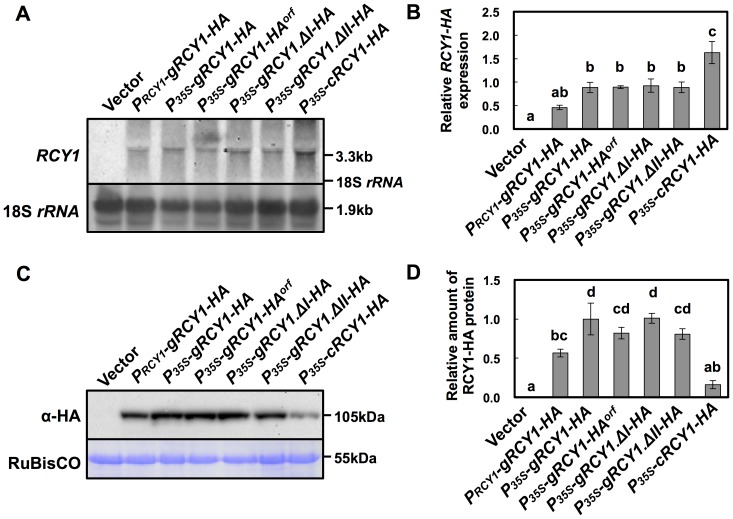
Detection of HA-epitope-tagged RCY1 protein and *RCY1* transcript in *N. benthamiana*leaves transiently expressing a series of *RCY1-HA*constructs under control of the *RCY1* or CaMV 35S promoters. *RCY1* transcripts in *N. benthamiana* leaves agro-infiltrated with*P_RCY1_-gRCY1-HA*, *P_35S_-gRCY1-HA*, *P_35S_-gRCY1-HA^orf^*, *P_35S_-gRCY1.ΔI-HA*, *P_35S_-gRCY1.ΔII-HA*, or *P_35S_-cRCY1-HA* were detected by northern hybridization. pRI201-AN (Vector) was used as an empty-vector control for agro-infiltration. As an internal control for RNA sample quantities, 18S *rRNA* is shown (A). Relative amounts of *RCY1*transcripts in each line were measured by quantitative RT-PCR. *EFα*gene expression was used as a standard for normalization of *RCY1*expression (B). HA-epitope-tagged RCY1 protein (α-HA) in *N. benthamiana* leaves transiently expressing *P_RCY1_-gRCY1-HA*, *P_35S_-gRCY1-HA*, *P_35S_-gRCY1-HA^orf^*, *P_35S_-gRCY1.ΔI-HA*, *P_35S_-gRCY1.ΔII-HA*, or *P_35S_-cRCY1-HA* was immunologically detected using anti-HA monoclonal antibody. As an internal control for protein sample quantities, the large subunit of RuBisCO was visualized by staining with CBB (C). RCY1-HA protein amounts in each line were quantified by band intensity using Quantity One software. For all experiments, four independent plants transiently expressing each vector construct were analyzed (D). The averages of relative *RCY1* transcript amounts ±SE are shown in B and D. In A and C, representative photographs are shown. The size of each band and the position of 18S *rRNA* were shown at right side of the panels. Data were subjected to analysis of variance and treatment means were compared by Tukey's test. Different letters indicate a statistically significant difference in the relative amount of *RCY1* transcript (*n*  =  4, *P*<0.05).

**Figure 6:**
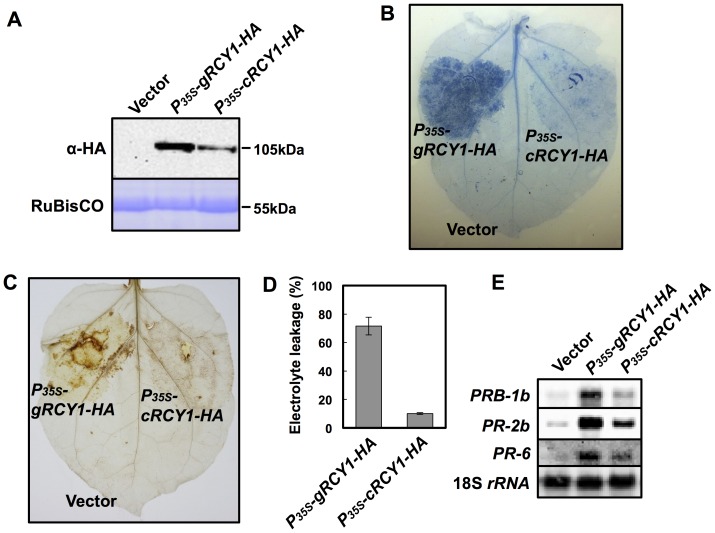
Activation of defense reaction in *N. benthamiana* leaves transiently expressing*P_35S_-gRCY1-HA* and *P_35S_-cRCY1-HA* under control of the CaMV 35S promoter. HA-epitope-tagged RCY1 protein (α-HA) (A) in *N. benthamiana* leaves transiently expressing *P_35S_-gRCY1-HA*, *P_35S_-cRCY1-HA*, or pRI201-AN (Vector) as an empty-vector control was immunologically detected using anti-HA monoclonal antibody. As an internal control for protein sample quantities, the large subunit of RuBisCO was visualized by staining with CBB. The size of each band was shown at right side of the panel. In *N. benthamiana* leaves transiently expressing *P_35S_-gRCY1-HA* or *P_35S_-cRCY1-HA*, hypersensitive response (HR) cell death was visualized by trypan blue staining (B), and H_2_O_2_ production was detected by DAB staining (C). To evaluate HR-cell death quantitatively, electrolyte leakage (D) in *N. benthamiana* leaves transiently expressing *P_35S_-gRCY1-HA*, *P_35S_-cRCY1-HA*, or empty-vector control was measured. Expression of the defense-related genes*PRB-1b*, *PR-2b*, and *PR-6* in *N. benthamiana* leaf tissue transiently expressing *P_35S_-gRCY1-HA*, *P_35S_-cRCY1-HA*, or the empty-vector control was analyzed by northern hybridization (E). As an internal control for RNA sample quantities, 18S *rRNA* was shown.

**Figure 7:**
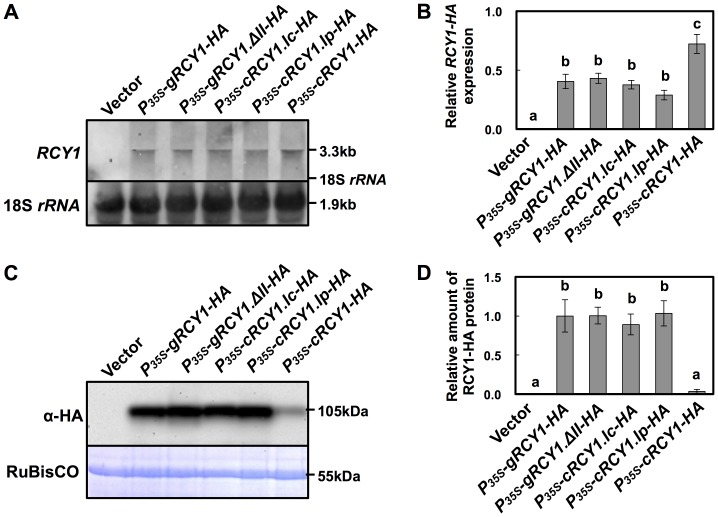
Detection of HA-epitope-tagged RCY1 protein and *RCY1* transcript in *N. benthamiana* leaves transiently expressing*RCY1-HA* constructs in which the *RCY1* introns were replaced with *COR15a* or *PRF3* introns. *RCY1* transcripts in *N. benthamiana* leaves agro-infiltrated with *P_35S_-gRCY1-HA*, *P_35S_-gRCY1.ΔII-HA*, *P_35S_-cRCY1.Ic-HA*, *P_35S_-cRCY1.Ip-HA*, or *P_35S_-cRCY1-HA* were detected by northern hybridization. pRI201-AN (Vector) was used as an empty-vector control for agro-infiltration. As an internal control for RNA sample quantities, 18S *rRNA* is shown (A). Relative amounts of *RCY1* transcripts in each line were measured by quantitative RT-PCR (B). HA-epitope-tagged RCY1 protein (α-HA) in *N. benthamiana* leaves transiently expressing *P_35S_-gRCY1-HA*,*P_35S_-gRCY1.ΔII-HA*, *P_35S_-cRCY1.Ic-H*A, *P_35S_-cRCY1.Ip-HA*, or *P_35S_-cRCY1-HA* was immunologically detected using anti-HA monoclonal antibody. As an internal control for protein sample quantities, the large subunit of RuBisCO was visualized by staining with CBB (C). RCY1-HA protein amounts in each line were quantified by band intensity using Quantity One software (D). For all experiments, four independent plants transiently expressing each vector construct were analyzed. The averages of relative amounts of *RCY1* transcript and protein ±SE are shown in B and D, respectively. In A and C, representative photographs are shown. The size of each band and the position of 18S *rRNA* were shown at right side of the panels. Data were subjected to analysis of variance and treatment means were compared by Tukey's test. Different letters indicate a statistically significant difference in the relative amount of *RCY1* transcript (*n*  =  4, *P*<0.05).

## Supporting Information

Figure S4 **Comparison of HA-epitope-tagged COR15a and PRF3 transcript and protein levels among** ***N. benthamiana*** **leaf tissues transiently expressing intron-containing genomic** ***COR15a*** **or** ***PRF3*** **or** ***COR15a*** **or** ***PRF3*** **cDNAs without introns.** *COR15a* (A) or *PRF3* (B) transcripts in *N. benthamiana* leaf tissues transiently expressing either the intron-containing genomic *COR15a*(*P_35S_-gCOR15a-HA*) or *PRF3* (*P_35S_-gPRF3-HA*), or the cDNAs for *COR15a* (*P_35S_-cCOR15a-HA*) or *PRF3* (*P_35S_-cPRF3-HA*) without introns, were detected by northern hybridization. pRI201-AN (Vector) was used as an empty-vector control. As an internal control for RNA sample quantities, 18S *rRNA* is shown. The size of each band and the position of 18S *rRNA* were shown at right side of the panels. COR15a (C) or PRF3 (D) protein amounts in each line were quantified by band intensity using Quantity One software. Four independent plants transiently expressing each vector construct were analyzed. The averages of relative COR15a-HA and PRF3-HA protein amounts ±SE are shown. The COR15a-HA and PRF3-HA proteins in leaf tissues of each line were also detected by immunoblotting (E). As controls, pRI201-AN (*Vector*), *P_35S_-gRCY1-HA*, and *P_35S_-cRCY1-HA*were agro-infiltrated into *N. benthamiana* leaves. As an internal control for protein sample quantities, the large subunit of RuBisCO was visualized by staining with CBB. In this experiment, 1/50 volume of total protein sample of leaf accumulating COR15a-HA against that of RCY1-HA and PRF3-HA was applied on the gel, since the level of COR15a-HA accumulation was essentially much higher than others. The size of each band and the position of RuBisCO large subunit were shown at right side of the panel.
(Tif)
