# Supplementary figures and images for: Role of Intron-Mediated Enhancement on Accumulation of an Arabidopsis NB-LRR Class R-protein that Confers Resistance to Cucumber mosaic virus
Source: PLoS One. 2014 Jun 10;9(6):e99041. doi: 10.1371/journal.pone.0099041 (PMC4051679; doi:10.1371/journal.pone.0099041)

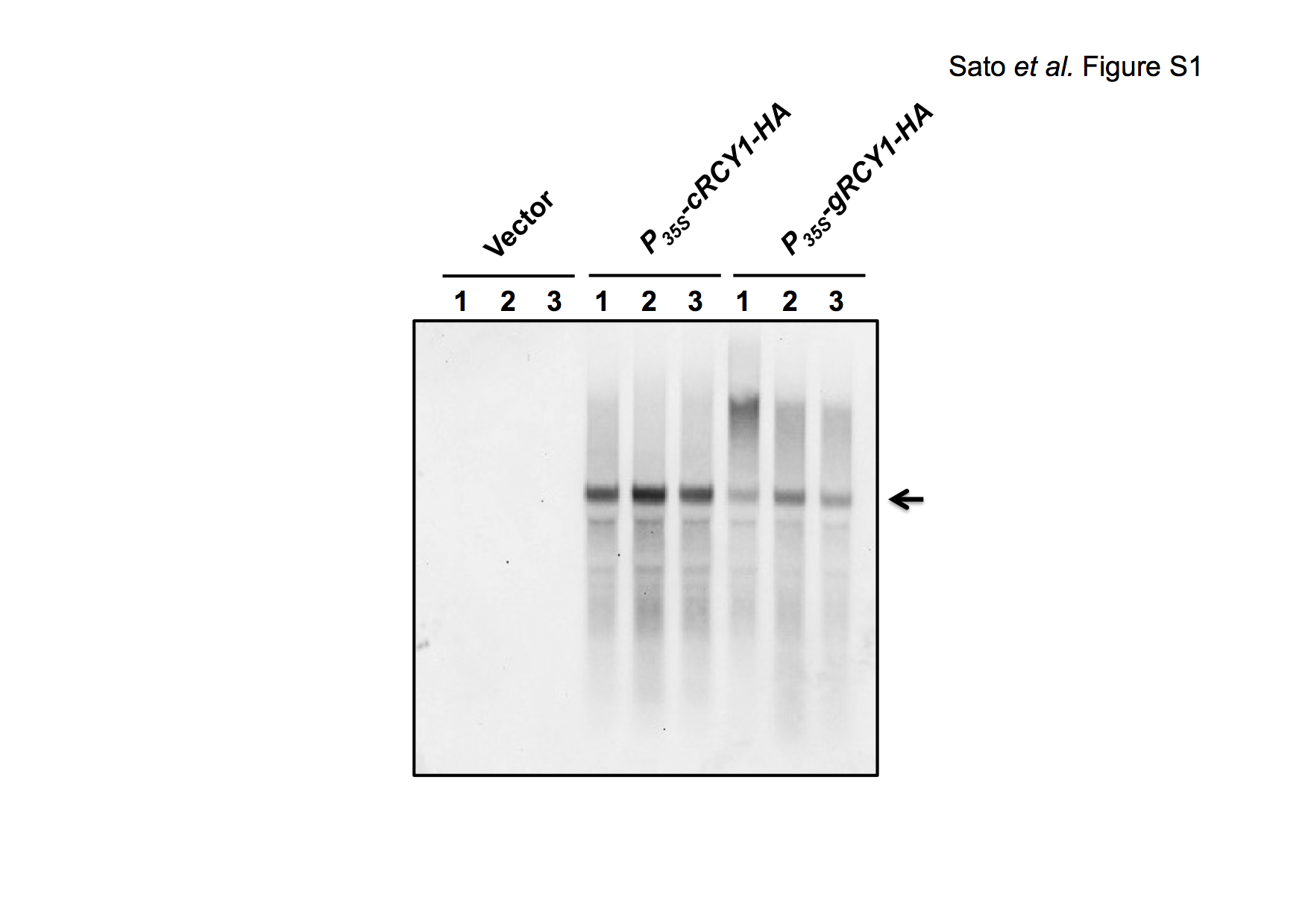

Supplement: Figure S1 — Detection of RCY1 transcripts in N. benthamiana leaves transiently expressing P35S-cRCY1-HA and P35S-gRCY1-HA . RCY1 transcripts in N. benthamiana leaves agro-infiltrated with the intron-containing genomic RCY1 coding region (P35S-gRCY1-HA), RCY1 cDNA without introns (P35S-cRCY1-HA), or pRI201-AN (Vector) as an empty vector control were detected by northern hybridization. Full-length RCY1 transcripts are indicated as bands marked by the arrow. RNA was extracted from three independent plants (1, 2, and 3) per vector-infiltrated plants. (TIFF) [file pone.0099041.s001.tiff]

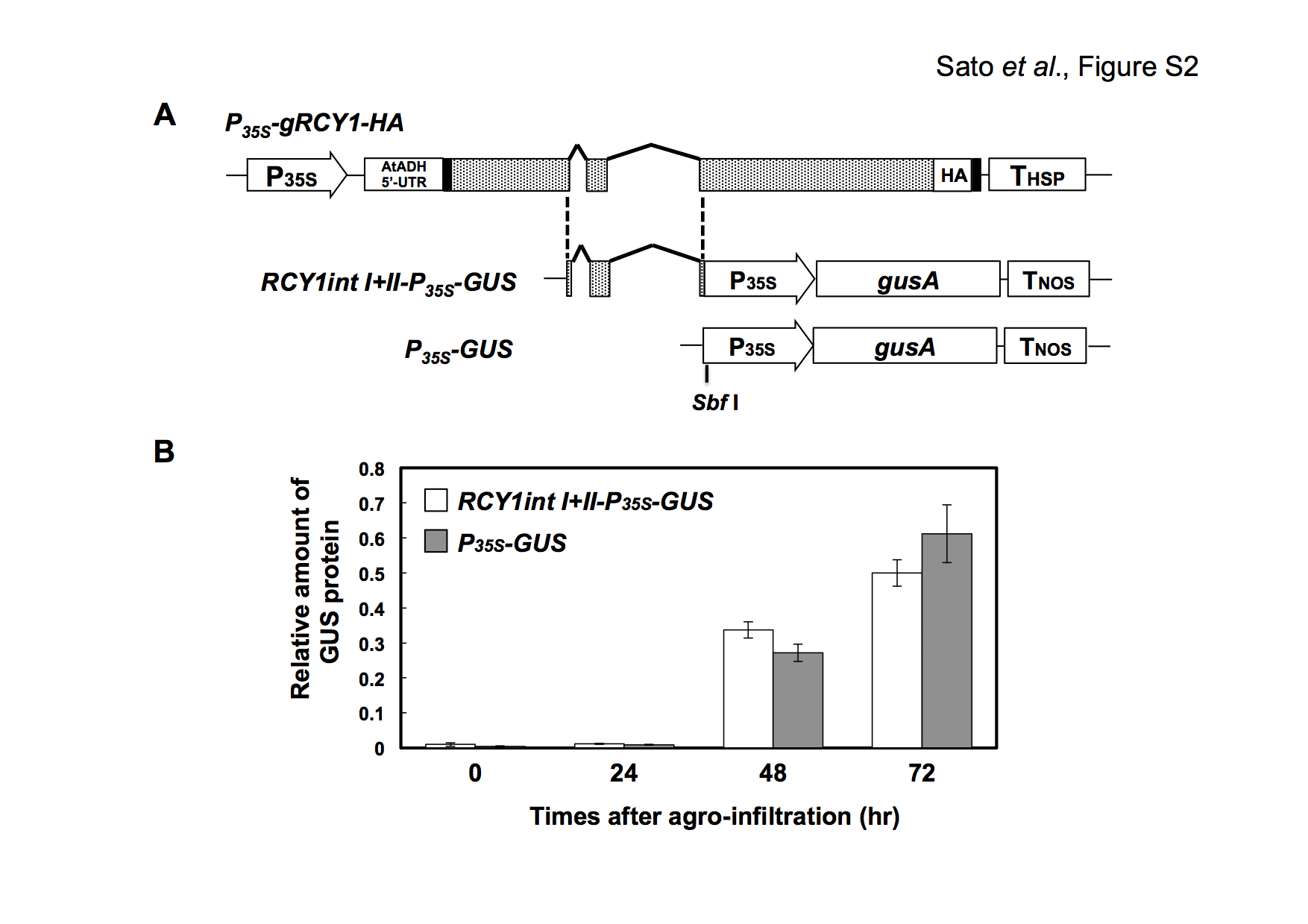

Supplement: Figure S2 — Assay for the enhancement of promoter activity by the RCY1 intron sequence. Schematic of the β-glucronidase (gusA)-coding vector constructs: P35S-GUS with gusA under control of the CaMV 35S promoter (P35S) and nopaline synthase terminator (TNOS); RCY1intI+II-P35S-GUS in which RCY1 intron sequences shown by the black fold lines, were inserted into SbfI site upstream of P35S of P35S-GUS (A). The position of insertion of the intron–containing fragment from P35S-gRCY1-HA into RCY1intI+II-P35S-GUS is indicated by dotted lines. Relative GUS protein quantities in N. benthamiana leaves transiently expressing P35S-GUS or RCY1intI+II-P35S-GUS were measured by ELISA at 0, 24, 48, and 72 h after agro-infiltration (B). Four independent plants transiently expressing each vector construct were analyzed. The averages of relative GUS protein amounts ±SE are shown in B. (TIFF) [file pone.0099041.s002.tiff]

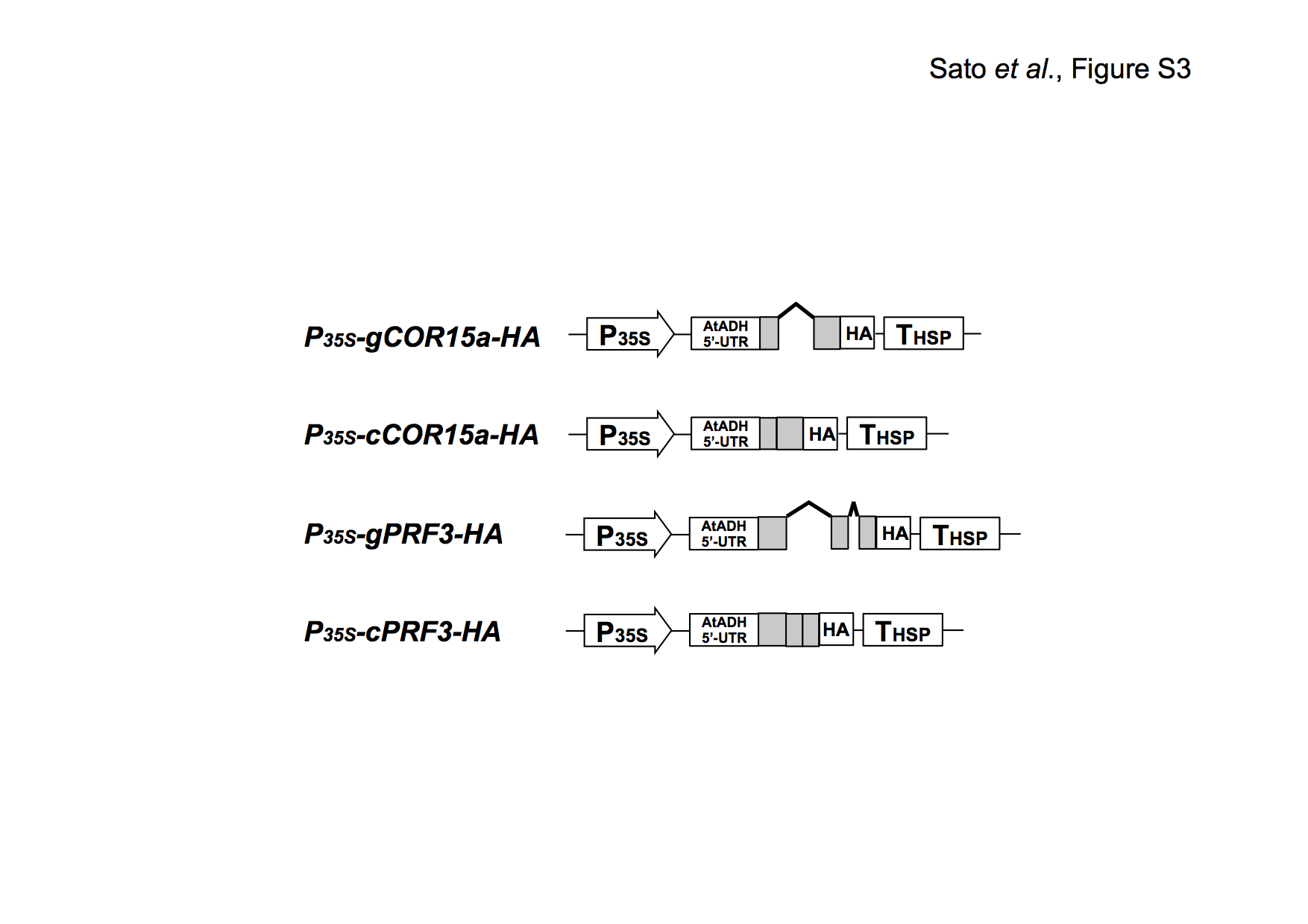

Supplement: Figure S3 — Schematic structure of the COR15a and PRF3 vector constructs under control of the CaMV 35S promoter. COR15a or PRF3 cDNA without introns but with HA-epitope tags (HA) at their 3′-ends were cloned between the CaMV 35S promoter with the 5′-UTR sequence of the Arabidopsis Alcohol Dehydrogenase gene (ADH5′-UTR) and Heat Shock Protein gene terminator (THSP) in the pRI201-AN binary vector. The resulting constructs were named P35S-cCOR15a-HA and P35S-cPRF3-HA, respectively. The COR15a- or PRF3-coding regions are indicated by gray boxes and the two splice junction sites are indicated by vertical lines in the boxes. Genomic COR15a or PRF3 tagged with HA at its 3′-end under control of the CaMV 35S promoter (P35S-gCOR15a-HA and P35S-gPRF3-HA) contains intron sequences indicated by the black dashed lines. (TIFF) [file pone.0099041.s003.tiff]

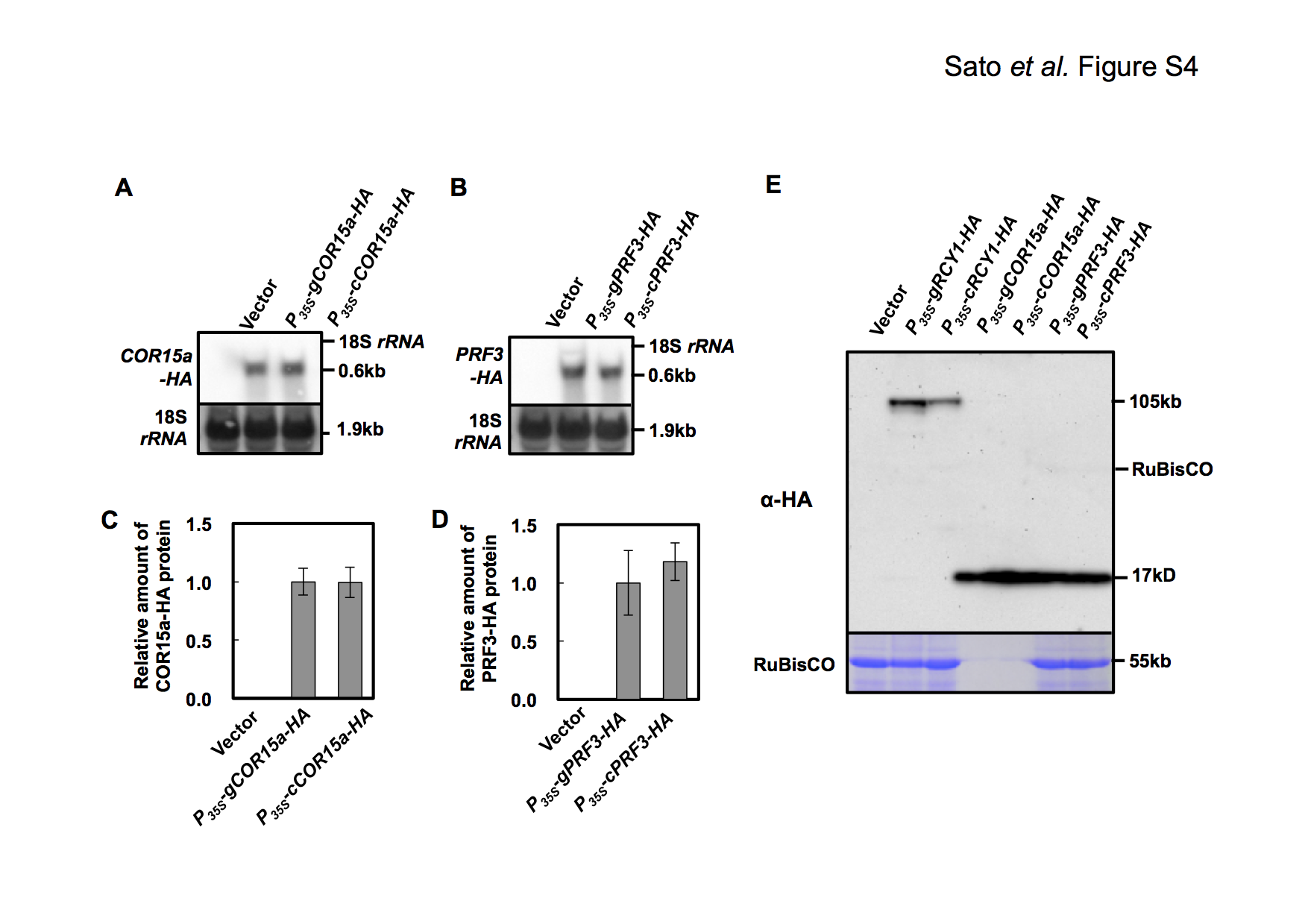

Supplement: Figure S4 — Comparison of HA-epitope-tagged COR15a and PRF3 transcript and protein levels among N. benthamiana leaf tissues transiently expressing intron-containing genomic COR15a or PRF3 or COR15a or PRF3 cDNAs without introns. COR15a (A) or PRF3 (B) transcripts in N. benthamiana leaf tissues transiently expressing either the intron-containing genomic COR15a (P35S-gCOR15a-HA) or PRF3 (P35S-gPRF3-HA), or the cDNAs for COR15a (P35S-cCOR15a-HA) or PRF3 (P35S-cPRF3-HA) without introns, were detected by northern hybridization. pRI201-AN (Vector) was used as an empty-vector control. As an internal control for RNA sample quantities, 18S rRNA is shown. The size of each band and the position of 18S rRNA were shown at right side of the panels. COR15a (C) or PRF3 (D) protein amounts in each line were quantified by band intensity using Quantity One software. Four independent plants transiently expressing each vector construct were analyzed. The averages of relative COR15a-HA and PRF3-HA protein amounts ±SE are shown. The COR15a-HA and PRF3-HA proteins in leaf tissues of each line were also detected by immunoblotting (E). As controls, pRI201-AN (Vector), P35S-gRCY1-HA, and P35S-cRCY1-HA were agro-infiltrated into N. benthamiana leaves. As an internal control for protein sample quantities, the large subunit of RuBisCO was visualized by staining with CBB. In this experiment, 1/50 volume of total protein sample of leaf accumulating COR15a-HA against that of RCY1-HA and PRF3-HA was applied on the gel, since the level of COR15a-HA accumulation was essentially much higher than others. The size of each band and the position of RuBisCO large subunit were shown at right side of the panel. (TIFF) [file pone.0099041.s004.tiff]
