# Supplementary figures and images for: Correction: Role of Intron-Mediated Enhancement on Accumulation of an Arabidopsis NB-LRR Class R-protein that Confers Resistance to Cucumber mosaic virus
Source: PLoS One. 2014 Sep 4;9(9):e107185. doi: 10.1371/journal.pone.0107185 (PMC4154769; doi:10.1371/journal.pone.0107185)

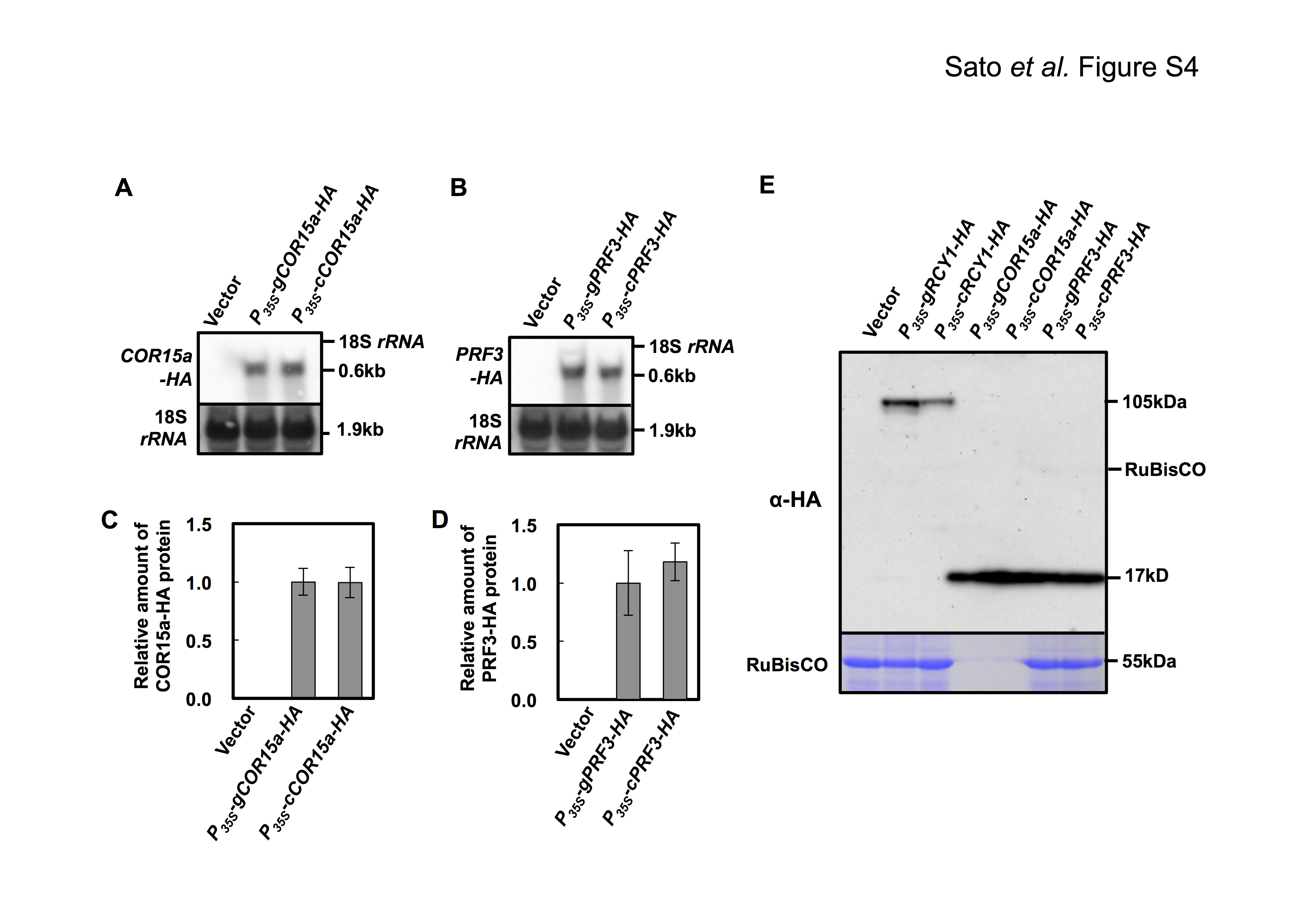

Supplement: Figure S4 — Comparison of HA-epitope-tagged COR15a and PRF3 transcript and protein levels among N. benthamiana leaf tissues transiently expressing intron-containing genomic COR15a or PRF3 or COR15a or PRF3 cDNAs without introns. COR15a (A) or PRF3 (B) transcripts in N. benthamiana leaf tissues transiently expressing either the intron-containing genomic COR15a(P35S-gCOR15a-HA) or PRF3 (P35S-gPRF3-HA), or the cDNAs for COR15a (P35S-cCOR15a-HA) or PRF3 (P35S-cPRF3-HA) without introns, were detected by northern hybridization. pRI201-AN (Vector) was used as an empty-vector control. As an internal control for RNA sample quantities, 18S rRNA is shown. The size of each band and the position of 18S rRNA were shown at right side of the panels. COR15a (C) or PRF3 (D) protein amounts in each line were quantified by band intensity using Quantity One software. Four independent plants transiently expressing each vector construct were analyzed. The averages of relative COR15a-HA and PRF3-HA protein amounts ±SE are shown. The COR15a-HA and PRF3-HA proteins in leaf tissues of each line were also detected by immunoblotting (E). As controls, pRI201-AN (Vector), P35S-gRCY1-HA, and P35S-cRCY1-HAwere agro-infiltrated into N. benthamiana leaves. As an internal control for protein sample quantities, the large subunit of RuBisCO was visualized by staining with CBB. In this experiment, 1/50 volume of total protein sample of leaf accumulating COR15a-HA against that of RCY1-HA and PRF3-HA was applied on the gel, since the level of COR15a-HA accumulation was essentially much higher than others. The size of each band and the position of RuBisCO large subunit were shown at right side of the panel. (Tif) [file pone.0107185.s004.tif]
